# Assessing the causal effects of environmental tobacco smoke exposure: a meta-analytic Mendelian randomization study

**DOI:** 10.1093/ntr/ntag047

**Published:** 2026-02-25

**Authors:** Benjamin Woolf, Skanda Rajasundaram, Janne Pott, Dipender Gill, Hannah M Sallis, Stephen Burgess, Marcus R Munafò

**Affiliations:** School of Psychological Science, University of Bristol, Bristol, BS8 1TU, United Kingdom; MRC Integrative Epidemiology Unit, University of Bristol, Bristol, BS1 5DS, United Kingdom; MRC Biostatistics Unit, University of Cambridge, Cambridge, CB2 0SR, United Kingdom; Moorfields Eye Hospital NHS Foundation Trust, London, EC1V 2PD, United Kingdom; MRC Biostatistics Unit, University of Cambridge, Cambridge, CB2 0SR, United Kingdom; Department of Epidemiology and Biostatistics, School of Public Health, Imperial College London, London, W12 0BZ, United Kingdom; MRC Integrative Epidemiology Unit, University of Bristol, Bristol, BS1 5DS, United Kingdom; Centre for Academic Mental Health, Population Health Sciences, Bristol Medical School, University of Bristol, Bristol, BS1 5DS, United Kingdom; MRC Biostatistics Unit, University of Cambridge, Cambridge, CB2 0SR, United Kingdom; British Heart Foundation Cardiovascular Epidemiology Unit, Department of Public Health and Primary Care, University of Cambridge, Cambridge, CB2 0BB, United Kingdom; School of Psychological Science, University of Bristol, Bristol, BS8 1TU, United Kingdom; MRC Integrative Epidemiology Unit, University of Bristol, Bristol, BS1 5DS, United Kingdom; University of Bath, Bath, BA2 7AY, United Kingdom

**Keywords:** environmental tobacco smoke, passive smoking, second-hand smoking, lung cancer, Mendelian randomization, ALSPAC

## Abstract

**Introduction:**

First-hand smoking is a major cause of global morbidity and mortality. Exposure to environmental tobacco smoke (ETS; “second-hand” or “passive smoking”) may also cause ill health, but establishing ETS as the cause is challenging, in part due to confounding and reverse causation.

**Methods:**

We applied Mendelian randomization (MR) to investigate the causal effects of ETS. We use four approaches to instrument ETS exposure: The first and second used an index individual’s parent’s genetically predicted smoking, independent of the index individual’s genetically predicted smoking to assess the effects of that parent’s smoking on the index individual. The third and fourth used one index individual’s parent’s genetically predicted smoking, independent of the other parent’s genetically predicted smoking to assess the effects of the first parent’s smoking on the second parent. We then meta-analyze the four MR approaches.

**Results:**

Our findings suggest a causal effect of genetically predicted ETS exposure on lung cancer and chronic obstructive pulmonary disease (*P*_FDR_ < .001 for both). We did not find evidence supporting an effect on hypertension, depression, coronary heart disease, or stroke (*P*_FDR_ = 1.000 for all four non-respiratory outcomes).

**Conclusion:**

These results support existing public health measures to limit exposure to ETS.

**Implications:**

We assess the causal effects of environmental tobacco smoking (ETS; “second-hand smoking” or “passive smoking”) using a quasi-experimental method, Mendelian randomization, which is more robust to confounding than conventional epidemiological methods.

To study the effects of ETS exposure, we used an index individual’s parent’s or spouses’ genetically predicted smoking, independent of either the index’s or the other parent’s genetically predicted smoking when assessing the effect of that parent’s smoking on the index individual or other parent, respectively. We then meta-analyze the effects of different relatives.

This study extends the Mendelian randomization paradigm to assess ETS by examining the effect that one relative has on the other relative, independent of the other relative’s smoking. In doing so, it adds a unique source of evidence that triangulates with prior research to indicate an effect of ETS exposure on lung cancer and chronic obstructive pulmonary disease.

## Introduction

Cigarette smoking is associated with numerous adverse health outcomes, ranging from lung cancer to depression.^1–7^ Many associations between cigarette smoking and adverse health outcomes have been replicated for environmental tobacco smoke exposure.^8–12^ Environmental tobacco smoke exposure (ETS, also referred to as “passive smoking” or “second-hand smoking”) occurs when an individual breathes in the cigarette smoke from another smoker. Establishing the specific causal effects for ETS exposure separate from first-hand smoking is challenging for various reasons. People more exposed to second-hand smoke may also be more exposed to various adverse socioeconomic risk factors and other confounders. People in poor health may be more likely to first-hand smoke and thus socialize with other smokers, leading to increased second-hand smoke exposure.

In addition to confounding and reverse causation, the tendency of individuals to choose friends with similar smoking habits,^13^ may also induce collider bias in studies of ETS exposure. Any observed association with ETS exposure could be a consequence of someone selecting friends with similar smoking habits and is thus a direct effect of cigarette smoking. This type of collider bias is difficult to account for in observational analyses of socially transmissible phenotypes.^14^ Many ETS studies are also at risk of differential recall bias.^15^ It is difficult to study the specific effects of exposure to ETS using many quasi-experimental methods because most interventions targeting ETS (eg, banning smoking in indoor public spaces) may influence the rate of first-hand smoking (eg, by reducing the rate that someone might smoke). The triangulation of evidence paradigm attempts to support causal inference in the absence of a perfectly unbiased estimate by comparing estimates from different designs with different biases.^16^ Since many of the aforementioned biases are present across much of the literature, studies using other methods are warranted.

Mendelian randomization (MR) leverages the random allocation of genetic variants at conception, analogous to random allocation in a randomized controlled trial,^17^^,^^18^ to reduce bias due to confounding: In the same way as treatment groups in a randomized trial are similar at the point of trial recruitment, groups of individuals with different genotypes should be similar to each other at conception.^19^ Any differences between individuals with and without a genetic variant should represent downstream consequences of the genetic variant. Furthermore, because germline genetic variants are fixed at conception, MR reduces susceptibility to reverse causality.

Thus, under specific assumptions, MR can strengthen causal inference. In brief, the three core assumptions are that the genetic variants robustly associate with the exposure of interest, that there are no variant–outcome confounders; and that the variants influence the outcome only via the exposure of interest.^20^ The first assumption can be tested empirically, the second follows from Mendel’s Law of Independent Assortment,^21^ and the third can be interrogated with sensitivity analyses. Introductory texts to the MR paradigm can be found in the following citations.^17–20^^,^^22^ Given that the MR assumptions are very different from those made in alternative study designs used to investigate the health effects of ETS, MR can help in triangulating the health effects of ETS.

**Figure 1:**
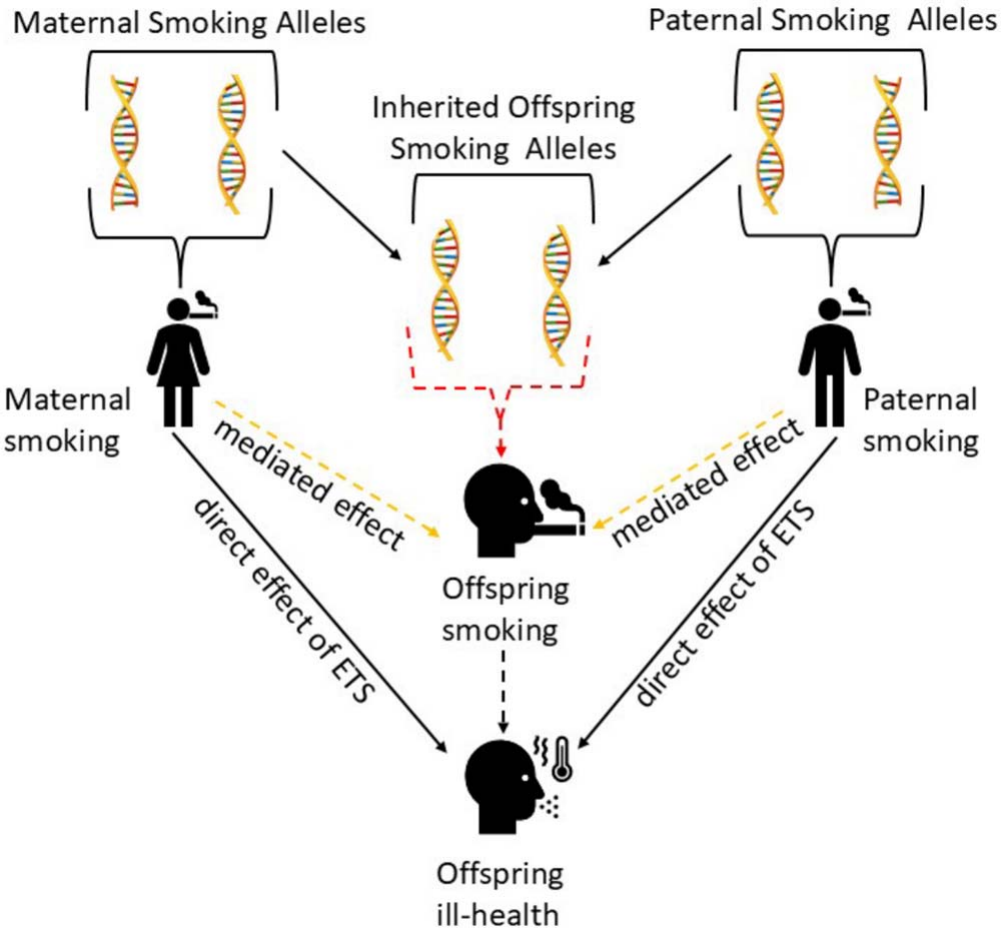
Diagram illustrating multivariable proxy gene-by-environment Mendelian randomization.

We therefore use MR to explore the causal effects of ETS exposure among relatives on a range of health outcomes. Since half of the variation in an individual’s genotype is shared with a given parent, an individual’s own genotype can be used as an imperfect proxy of their parents’ genotype when parental genetic data is unavailable. MR studies that proxy the parental genotype with the offspring’s genotype when only parental phenotypic data are available (eg, via offspring-reported questionnaire) are called “proxy gene-by-environment” MR studies.^23^ Conditional on parental genotype, the inheritance of parental variants should be independent of environmental confounders, and so this approach does not require explicit modeling of environmental determinants of parental smoking or offspring outcomes. However, if the effect of the variants on the index individual’s phenotype due to genetic inheritance is not accounted for (red dashed line in Figure 1), the resulting proxy gene-by-environment MR estimate will be biased.^24^^,^^25^

Proxy gene-by-environment MR has previously been used to examine the effects of maternal smoking during pregnancy on her offspring’s health at birth in samples like the UK Biobank (UKB) where maternal genetic information is not available.^23^ In a proof-of-concept study in UKB, Yang et al. used a variant in the nicotine metabolism gene *CHRNA5* known to influence smoking behavior to triangulate an observed effect of maternal smoking during pregnancy on lower birthweight, observed in the observational literature, among UK Biobank participant’s whose mothers smoked, Their conclusion affirming an association was strengthened by the failure to detect an effect among non-smoking mothers.

**Figure 2:**
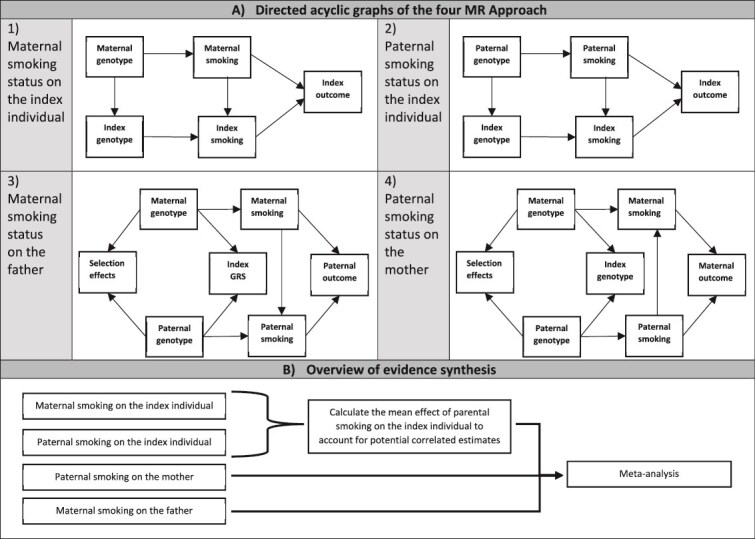
Description and overview of the study design and four Mendelian randomization approaches. *Note:* We omit potential pleiotropic and phenotypic confounding effects in the directed acyclic graphs in (A) to emphasize possible biases that could occur due to the genetic covariance between family members. For simplicity, we have also omitted possible bidirectional effects between parental and index individual smoking or between both parents, because such bidirectional effects would not be included in the direct effects being estimated. However, we note that the direct effects exclude effects of parental smoking mediated by the offspring’s/other parent’s smoking on the respective individuals’ outcomes. In (B), we only include the mean estimate from the two approaches assessing the effects of parental smoking on the index individual to account for the estimates being potentially correlated.

Here, we use a multivariable extension of proxy gene-by-environment MR to assess the direct effect of parental genetically predicted smoking independent of offspring genetically predicted smoking. We used four approaches: The first two approaches tested the direct effects, respectively, of maternal and paternal smoking on their offspring’s health outcome (illustrated by Figures 1 and 2A1 and A2), while the other two approaches examined the direct effects parents have on each other’s outcomes (illustrated in Figure 2A3 and A4). We used large-scale publicly available genetic data to evaluate the effect of ETS on six outcomes: lung cancer, chronic obstructive pulmonary disease (COPD), stroke, coronary heart disease, hypertension, and depression. We conducted a risk of bias evaluation, after which independent estimates were meta-analyzed to provide a pooled estimate of the effect of ETS on each outcome.

## Methods

### Summary and overview of methods

This study used multivariable Mendelian randomization (MVMR) to evaluate the effects of ETS. To do this, we applied four approaches that use MR to assess the effect of exposure to a relative’s smoking and therefore ETS exposure (Figure 2A). Specifically, we examine:

- The direct effect of genetically predicted maternal smoking on offspring outcomes, independent of genetically predicted offspring smoking (Figure 2A1).
- The direct effect of genetically predicted paternal smoking on offspring outcomes, independent of genetically predicted offspring smoking (Figure 2A2).
- The direct effect of genetically predicted maternal smoking on paternal outcomes, independent of genetically predicted paternal smoking (Figure 2A3).
- The direct effect of genetically predicted paternal smoking on maternal outcomes, independent of genetically predicted maternal smoking (Figure 2A4).

We implemented these analyses using summary data from the UK Biobank, FinnGen, Avon Longitudinal Study of Parents and Children (ALSPAC), and relevant disease-specific consortia.^26–35^ The UK Biobank was the only data source measuring parental disease phenotypes. To qualify as an outcome, parental disease phenotypes had to satisfy three criteria: (1) be matched with a measured outcome in the participant’s generation for the UK Biobank and other data sources used here, (2) have a non-zero number of events in the UK Biobank for both sexes in the parental generation, and (3) have previously been identified as being associated with ETS exposure^8–12^^,^^36^^,^^37^ and thus plausibly contribute to the disease burden attributable to ETS.^38^ Lung cancer, COPD, stroke, coronary heart disease, hypertension, and depression met these criteria.

To increase statistical power, we meta-analyzed independent estimates from our four approaches (Figure 2B). Further, we investigated the robustness of the three core MR assumptions using weak instrument– and pleiotropy-robust estimators, falsification tests for pleiotropy, and negative controls for population structure and genotype-chip-based selection bias. We performed leave-one-out analyses in which we combined estimates from each of the three of the four approaches listed above, in turn, and the GRADE evaluation to assess the overall conclusions from the meta-analyses.

The remainder of the Methods section and the Supplementary Methods provide a more in-depth explanation of the precise approaches used.

### Overview of multivariable gene-by-environment Mendelian randomization approaches

Traditional “proxy gene-by-environment” MR designs use the index individual’s genotype as a proxy of a parent’s genotype, thus estimating effects of genetically predicted parental smoking in settings where parental phenotypic data are available but parental genetic data are not. This is possible because offspring inherit half of each parent’s variants, so these variants also affect the respective parental phenotype. As the approach uses the offspring’s genetic variants, an advantage of its two-sample MR implementation is that outcome data can be extracted from conventional genome-wide association studies (GWASs), which are much larger than current studies with genetic data on multiple relatives.

However, the use of inherited variants can bias proxy gene-by-environment MR through the effect that variants have on the index individual’s outcome that is not mediated by parental phenotype (red dashed line in Figure 1).^24^ We circumvent this by including the index individual’s genetically predicted smoking behavior as a covariate in an MVMR model. Multivariable MR estimates are interpreted as the direct effect of the exposure independent of the covariate phenotype. Multivariable MR has therefore been used to attenuate postulated biases in MR estimates.^39–44^ We call our application of MVMR “multivariable gene-by-environment MR.” We note that although the index’s smoking in Figure 2A1 is a collider on the parental smoking–offspring outcome association, adjustment using MVMR does not introduce collider bias.^39^^,^^42–44^

In the first approach, we perform multivariable gene-by-environment MR by including both maternal genetically predicted smoking and the index individual’s genetically predicted smoking as exposures in the model. This assesses the direct effect of genetically predicted maternal smoking on the index individual’s outcomes, independent of genetically predicted offspring smoking (Figure 2A1).

In the second approach, we include paternal and offspring genetically predicted smoking as exposure phenotypes. This approach assesses the direct effects of genetically predicted paternal smoking on the index individual’s outcomes independent of the index individual’s genetically predicted smoking behavior (Figure 2A2).

The third and fourth approaches include both maternal and paternal genetically predicted smoking as the exposures in a multivariable gene-by-environment MR model. The third approach investigates paternal outcomes and assesses the direct effects of maternal genetically predicted smoking on paternal outcomes, independent of genetically predicted paternal smoking (Figure 2A3). The fourth approach investigates maternal outcomes and assesses the direct effects of genetically predicted paternal smoking on maternal outcomes independent of genetically predicted maternal smoking (Figure 2A4).

The last two approaches leveraged GWASs (described in the next section and the Supplementary Methods) for maternal smoking and paternal outcomes to explore the effects that one parent has on the other parent. Here, the likely source of bias is assortative mating, where parents tend to partner with people who have a similar smoking status. A naive association between one parent’s smoking and the other’s outcomes could represent the combined effects of first-hand smoking and assortative mating.^13^ Using similar logic to the first two approaches, assortative mating can be controlled for by adjusting for the other parent’s genetically predicted smoking in a multivariable gene-by-environment MR model.

An explanation of how to implement summary data MVMR can be found in the Supplementary Methods.

### Data sources

Data on maternal smoking and index individual smoking were used publicly available GWAS summary statistics using a sub-sample of genetically unrelated European ancestry participants in the UKB (*n* ~ 400 000), which have been described elsewhere.^30^^,^^45^ Variants associated with paternal smoking were also selected using a multivariate European ancestry UKB GWAS.^46^^,^^47^ Since this GWAS made strong parametric assumptions,^48^ variant–paternal smoking associations used in the analysis were estimated in ALSPAC (*n* = 5766, see Supplementary Methods and^49^^,^^50^ for details). Both parental smoking GWASs measured parental smoking status; index individual smoking measured the lifetime smoking index (see Supplementary Methods for more details).

The index individual outcome GWASs combined the UKB with FinnGen r10 and independent but demographically similar samples of the same trait taken from available genetic consortia and meta-analyses. These include the International Lung Cancer Consortium for lung cancer, Psychiatric Genetic Consortium for depression, the European sub-sample of the Global Biobank Meta-Analysis Initiative for COPD, CARDIoGRAMplusC4D for coronary heart disease, and the European ancestry sub-sample of GIGASTROKE for stroke. This resulted in 20 359 cases and controls for 810 746 lung cancer; 58 559 cases and 937 358 controls for COPD; 170 756 cases and 329 443 controls for depression; 1 234 808 cases and 1 308 460 controls for stroke; 239 785 cases and 1 355 114 controls for coronary heart disease; and 242 724 case and 649 418 controls for hypertension. Parental outcome data were also taken from European ancestry UKB GWASs of index individual reported maternal and paternal outcomes (see Supplementary Figure S1). To be consistent with the CARDIoGRAMplusC4D study, we use the term “coronary heart disease” when describing the results of our meta-analysis, although the outcome used in the UK Biobank questionnaire of parental disease outcomes used the less precise term “heart disease.”

The data sources, and number of participants and variants, included in each analysis for the four MR approaches are summarized in Supplementary Figure S1. Additional details such as information about the data sources and statistical methods used in the MR approaches can be found in the Supplementary Methods and at the following references.^26–35^

### Instrument construction

For each of the MR approaches, we created a list of variants that were associated with either of the smoking phenotypes used in the analysis at *P* < 5 × 10^−8^. We then clumped the list of variants (*r*^2^ **=** 0.001, kb = 10 000 using the European sub-sample of the 1000 Genomes Reference panel) after ranking them by the smallest *P*-value that they had with either exposure. The analysis of maternal smoking on the index individual (Figure 2A1) therefore used the maternal smoking and index individual smoking GWASs, which resulted in at most 129 variants. Similarly, the procedure resulted in 127 variants for the second approach (Figure 2A2) and 19 variants in the third and fourth approach (Figure 2A3 and A4). We used the false discovery rate inverse quantile transformation Winner’s Curse correction to account for possible Winner’s Curse in the maternal and index individual smoking GWASs.^51^

### Meta-analysis of MR approaches

A literal interpretation of our MR estimates requires strong assumptions, such as that ETS exposure does not vary across the life course.^52^ One can alternatively treat MR as a test of a causal null hypothesis.^53^^,^^54^ In such settings, a sufficient condition to interpret the direction (but not size) of the effect is that the direction of the variant’s association with the exposure is constant over the life course.^54^ It has been similarly argued that meta-analyses of randomized controlled trial should be used to “determine whether or not some type of treatment - tested in a wide range of trials - produces of an effect,” rather than “provide exact quantitative estimates.”^55^ Power to reject the joint null hypothesis, that exposure to another individual’s genetically predicted smoking does not increase an index individual’s disease risk, can therefore be increased by a quantitative meta-analysis of the four MR estimates.

Since our aim is to test the joint null hypotheses, we used a common effect meta-analysis (of the IVW MR estimates) as our primary analysis in line with Peto and others.^56^^,^^57^ The summary measure of the association in meta-analyses was the log odds in the outcome per genetically predicted standard deviation increase in exposure to ETS. We accounted for a potential correlation between the approaches assessing parental smoking and the index individual outcomes (Supplementary Table S1) by meta-analyzing the unweighted average of their effect estimates and 95% CI of potentially correlated pair.^58^^,^^59^

### Sensitivity and additional analyses

We ran five sensitivity analyses for the MR approaches. First, as a positive control, we ran a univariable MR analysis of the index individual’s own cigarette smoking on their own outcomes. The positive control also helps show that sufficient statistical power is present to detect an effect on the outcomes^54^ where one is known to exist. This is necessary because there are currently no existing methods for implementing a power calculation for MVMR. Second, we used hair color as a negative control outcome for residual population structure.^60^ Third, the UK Biobank genotyping chip used depended on participants’ lung function. Since smoking impacts on lung function, individuals who smoked were preferentially sampled into the UKBileve sub-sample that used a different genotyping chip. This could potentially induce selection bias. However, our previous research found that MR estimates of first-hand smoking are little changed after accounting for this selection effect.^44^ Since any bias should be larger for first-hand smoking than ETS, this indicates that there should be little bias in the present study. We follow existing guidance and contrast MR estimates using chip-adjusted and non-chip-adjusted GWASs for lung function–related phenotypes as a negative control analysis.^45^ Fourth, we assess pleiotropy using the MR-Egger intercept, Cochrane *Q* statistic, and comparing the homogeneity in three pleiotropy robust estimators described in the Supplementary Methods. Finally, we assessed weak instrument bias using the conditional *F* statistics and four weak instrument robust estimators described in the Supplementary Methods.

Heterogeneity in the meta-analysis was assessed using the *I*^2^ and τ ^2^ statistics, and we used the random-effects model as a secondary estimator. We also used a leave-one-out sensitivity analysis to explore the potential effect of, and robustness to, outliers in the primary analysis. Finally, we control for multiple testing across the six outcomes using the Benjamini and Hochberg correction.^61^

### Certainty assessment for the meta-analysis

Uncertainty was evaluated for each outcome using the Grading of Recommendations, Assessment, Development and Evaluations (GRADE) approach for the primary estimate (ie, common effect meta-analysis).^62^^,^^63^ Since our estimates are from theoretically low-risk-of-bias natural experiments, we follow Kim and colleagues and start all meta-analyses at high certainty and then downgrade them where appropriate.^64^ To aid with the certainty evaluation, we developed a bespoke risk of bias tool described in the Supplementary Methods. Although we otherwise adhere to the GRADE guidelines,^65^ we acknowledge that judging imprecision, heterogeneity, and publication bias is, in part, subjective.

## Results

### Results of the meta-analyses

**Figure 3:**
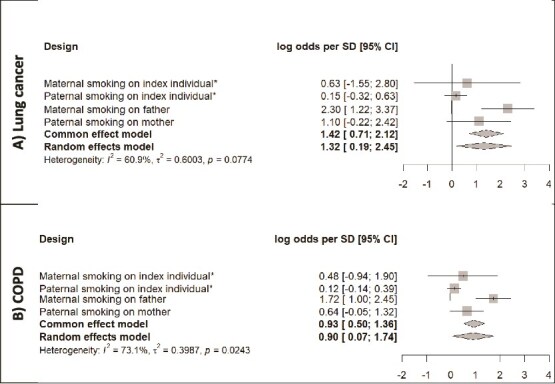
Forest plots of the meta-analysis of MR estimation approaches for lung cancer and chronic obstructive pulmonary disease (COPD). Results have units of log odds of the outcomes per standard deviation in relative’s genetically predicted smoking. Mendelian randomization estimates were derived using the inverse-variance weighted Mendelian randomization method. *Instead of including the MR estimates of maternal and paternal genetically predicted smoking on the index individual in the meta-analysis, we instead included their unweighted average to account for these MR estimates not being independent (see Supplementary Table S1).

**Figure 4:**
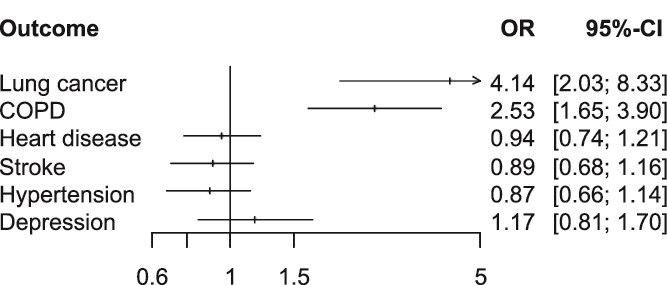
Results of the meta-analysis of MR estimation approaches for the six outcomes. Odds ratios (ORs) represent the multiplicative increase in the odds of the outcome per standard deviation increase in a relative’s genetically predicted smoking.

We found evidence that greater genetically-predicted exposure to ETS associates with an increase in the odds of developing lung cancer (odds per SD = 4.14 [95% CI: 2.03 to 8.33, *P*_FDR_ < .001, *I*^2^ = 61%], Figure 3A) and chronic obstructive pulmonary disease [COPD] (odds per SD = 2.53 [95% CI: 1.65 to 3.90, *P*_FDR_ < .001, *I*^2^ = 73%], Figure 3B). We did not find clear evidence of association for other outcomes (heart disease = 0.94 [95% CI: 0.74 to 1.21, *I*^2^ = 0%, *P*_crude_ = .652, *P*_FDR_ = 1], stroke 0.89 [95% CI: 0.68 to 1.16, *I*^2^ = 0%, *P*_crude_ = .396, *P*_FDR_ = 1], hypertension = 0.87 [95% CI: 0.66 to 1.14, *I*^2^ = 0%, *P*_crude_ = .319, *P*_FDR_ = 1], depression = 1.17 [95% CI: 0.81 to 1.70, *I*^2^ = 0%, *P*_crude_ = 0.388, *P*_FDR_ = 1]). The forest plots for all of the meta-analyses of results are presented in Figure 4 and Supplementary Figure S4.

### Results of sensitivity and additional analyses

Our positive control analysis (assessing the effects of first-hand smoking) found evidence of an association between index individual smoking and all index individual outcomes in a univariable MR analysis (Supplementary Table S2). Neither the hair color negative control analysis nor the comparison between the chip-adjusted GWASs and no-chip-adjusted GWAS MR estimates indicated evidence of bias (Supplementary Table S4 and Supplementary Table S5, respectively). The MR Egger intercepts generally did not indicate the presence of pleiotropy, and pleiotropy robust estimators had relatively homogeneous estimates in each analysis, but the Cochran *Q* statistic did find evidence of heterogeneity between variant-specific effects—especially in the parental smoking on index outcome analyses (Supplementary Table S2). The conditional instrument strength was very low in all analyses, while weak instrument robust estimators were homogeneous; they were also highly imprecise (Supplementary Table S2). A risk of bias evaluation for each approach stratified by outcome can be found in Supplementary Table S3 and the Supplementary Results.

The leave-one-out analysis indicated that excluding the “Maternal smoking on paternal outcomes” approach results in smaller estimates (which are only indicatively significant for COPD) for the effect of ETS on lung cancer and COPD (Supplementary Figure S2).

#### Certainty of evidence in meta-analysis

The GRADE evaluation of the certainty of evidence for our meta-analyses can be found in Table 1. We downgraded all outcomes to moderate certainty due to unclear risk of bias from conditional weak instruments. In addition, lung cancer and COPD were downgraded to low certainty due to heterogeneity in the leave-one-out analyses.

**Table 1:** GRADE evaluation of the certainty in the evidence for our six outcomes

GRADE domain	Lung cancer and chronic obstructive pulmonary disease	Stroke, cardiovascular disease, hypertension, depression
**Overall GRADE evaluation**	Low certainty	Moderate certainty
**Heterogeneity and inconsistency**	Heterogeneity in the leave-one-out analysis in Supplementary Figure S2 (downgrade).	No heterogeneity in the primary meta-analysis or leave-one-out (no downgrade).
**Risk of bias**	A full risk of bias evaluation can be found in Supplementary Table S3. All designs were downgraded to unclear risk of bias due to very low conditional *F* statistics. Although the weak-instrument robust estimators had consistent estimates, they also tended to have wide confidence intervals. A downgrade to unclear risk of bias was therefore deemed appropriate to reflect the uncertainty in the estimates (downgrade).	
**Imprecision**	There are no currently existing power calculations for multivariable MR studies. We instead used the positive control analysis of first-hand smoking as a benchmark. This should ensure sufficient power to detect effects at least as large as first-hand smoking, although there may still be low power to detect much smaller effects (no downgrade).	
**Indirectness**	All studies estimate the effect of ETS in a population of adult Europeans (no downgrade).	
**Publication bias**	Publication bias occurs from research not included in a literature search. Since our meta-analysis was not conducted after a literature review publication bias should not occur (no downgrade).	

## Discussion

We have leveraged genetic data in a multivariable gene-by-environment MR framework to investigate the long-term effects of ETS on lung cancer, COPD, stroke, heart disease, hypertension, and depression. After accounting for multiple testing, we found evidence of an association between second-hand smoking and both lung cancer and COPD. According to the GRADE approach, these should be interpreted with low certainty because of heterogeneity in the leave-one-out analyses driven by the strong effect of maternal smoking on maternal outcomes. However, with moderate certainty, we did not find evidence of an association with heart disease, stroke, hypertension, or depression.

### Comparison with existing research

Since both first-hand smoking and ETS involve inhaling tobacco smoke, it is reasonable to expect the harms of first-hand smoking to generalize to ETS to some extent. Our results, on the one hand, triangulate with existing research providing evidence for a harmful causal effect of ETS. Consistent with the existing literature, our results support an effect of ETS on lung cancer^9^^,^^66^ and COPD^67–69^ and a null association between ETS exposure and blood pressure.^70^ However, previous meta-analyses of observational studies comparing people’s reported ETS exposure to outcome risk have found a significant dose–response relationship between time exposed to ETS and risk of depression, stroke, and heart disease.^11^^,^^71–77^ Even so, it has been claimed that the effects of ETS have been overestimated^78^^,^^79^ and so it is possible that the null results in the present study reflect a greater robustness to residual confounding, bias due to non-random selection of peers, and reverse causation.

Our null results should, however, be interpreted with due caution. Firstly, proxy gene-by-environment MR is less statistically powerful than traditional approaches.^80^ We were able to somewhat mitigate this issue through meta-analysis, which resulted in 75 000–1 358 000 cases, and over a million controls, for each outcome. Nevertheless, the estimates for first-hand smoking on the non-respiratory outcomes were much smaller than the estimates on lung cancer or COPD (Supplementary Table S2). As ETS is dispersed in the air, its effects on a per-cigarette basis should be even smaller than that of directly inhaled smoke. Environmental tobacco smoke might thus have subsyndromal effects on the non-respiratory disease outcomes explored here, such as a small reduction in depressive symptoms or blood pressure, which are difficult to detect with disease diagnosis data and compatible with our wide 95% confidence intervals. Since there are no robust methods for calculating power for MVMR analyses, it is challenging to formally investigate this limitation.

Moreover, the effect of smoke inhalation on lung cancer is cumulative over the life course, such that current and former smokers remain at higher risk than never smokers.^81^ For outcomes such as depression, however, smoking cessation may be followed by a recovery process that results in a convergence of disease risk for former smokers with never smokers over time.^82^ This could mask a short-term risk increase given a large lag between ETS exposure and outcome measurement. However, COPD risk also follows this trajectory, implying that it cannot completely explain the contrasting results for respiratory vs non-respiratory outcomes.^83^^,^^84^

### Strengths and limitations

Multivariable proxy gene-by-environment MR has several theoretical advantages over a conventional observational design. As biological relatives do not choose each other, the parent–offspring MR analyses should be more robust to potential biases due to the non-random selection of peer groups.^85^ Likewise, because genes are fixed at conception, genetically predicted smoking cannot be impacted by the amount of smoking in one’s environment. Thus, our MR estimates should be more robust to reverse causation than an observational study. In addition, using MVMR to control for the individual’s genetic liability to smoke should attenuate bias from the variant having multiple effects in the index individual. The variant could still have multiple effects in the parent, but these would have to be socially transmittable to impact on the index individual. Although smoking has been shown to be a highly pleiotropic phenotype,^86^ it is not clear how the identified socially transmittable pleiotropic phenotypes (eg, alcohol consumption) could cause COPD and lung cancer in other people independently of smoking.

As with all approaches to causal inference, our MR analysis makes strong assumptions that are not verifiable. Thus, multivariable proxy gene-by-environment MR analyses cannot provide definitive evidence for or against a causal effect and need to be triangulated with other approaches.^16^ However, the approach is informative within a triangulation framework, since it provides a radically different type of evidence from other designs. The use of summary-level data necessitates making parametric assumptions like linearity and no effect modification. Multivariable MR also requires that we have conditionally strong instruments for the phenotypes. The low conditional *F* statistics (Supplementary Table S2) are a major factor driving the low certainty in our study results. Since variant–phenotype associations are likely to be similar between parents and their offspring for all biologically plausible phenotypes, conditional weak instrument bias is likely to be a major caveat for any plausible application of the multivariable proxy-MR design. However, it is not clear that there is a better alternative MR design that can be implemented with existing data.

**Figure 5:**
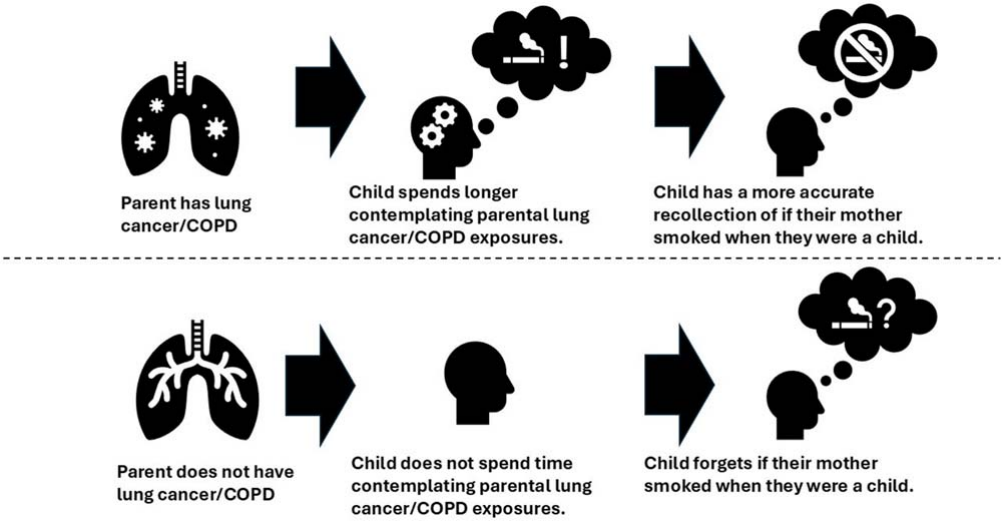
Illustration of respiratory outcome-related recall bias in maternal smoking.

A second caveat is the large apparent effect of ETS on lung cancer and COPD, which our meta-analyses implausibly imply are only marginally smaller per standard deviation (SD) than the effects of first-hand smoking (Supplementary Table S2). The forest plots (Supplementary Figure S4) and leave-one-out analysis (Supplementary Figure S2) imply that this is driven by the design assessing the effects of maternal smoking on paternal outcomes. Supplementary Table S6 provides possible explanations we are aware of. The explanation that best accounts for the why the extreme effects are specific to the effects of maternal smoking on paternal respiratory outcomes is outcome-related recall bias in the measure of maternal smoking (Figure 5): Individuals whose parents had lung cancer or COPD might more accurately remember if their mother smoked when they were children than those who did not, resulting in an inflated estimate when there is a true effect.^15^ Recall bias is unlikely to impact the designs using paternal smoking because, in contrast to maternal smoking, which was reported retrospectively in the UK Biobank, paternal smoking was measured prospectively in ALSPAC. COPD and lung cancer are widely known to be caused by smoking but less so for outcomes such as depression. Hence, a parent developing a non-respiratory outcome would be less likely to induce differential recall in parental smoking status. Outcome-related recall bias in maternal smoking will only inflate estimates from valid instruments when there is a true effect and thus cannot result in false positives^41^ (Supplementary Figure S3). On balance, therefore, the large estimates cannot be readily explained away as false-positive findings. Equally, we reiterate that our MR estimates do not represent the quantitative impact of a shift in smoking behavior in practice.

Finally, we have assumed that any effect detected reflects the life-course effect of ETS. This assumption is reasonable for COPD and lung cancer, but the effect of maternal smoking on her offspring may reflect in utero or other biological effects (eg, due to breast milk) for other outcomes.

### Conclusion

We developed a multivariable proxy gene-by-environment MR approach to assess the causal effect of ETS exposure on lung cancer, COPD, stroke, heart disease, hypertension, and depression. Our findings suggest that there is a causal relationship between exposure to ETS and the development of lung cancer and COPD. Triangulating our findings with existing research, our study strengthens the evidence supporting existing public-health interventions to reduce people’s exposure to ETS. However, we did not find evidence of a causal effect of ETS on heart disease, stroke, depression, or hypertension. Until it becomes possible to conduct a better powered MR study, the null associations observed between ETS and non-pulmonary conditions should be interpreted cautiously.

## Supplementary Material

Supplementary_Material_ntag047

## Data Availability

The data that were used in the primary analyses of this study are publicly available from the MRC-IEU OpenGWAS platform. The R code used in the manuscript, and GWASs created for this project, are available from https://doi.org/10.17605/OSF.IO/BKYXT. This project was conducted using UK Biobank application no. 15825. UK Biobank is open to bona fide researchers anywhere in the world. The data used in this study are available via application directly to ALSPAC (proposal number B4180). Access is subject to an approved proposal and payment of a data access fee. Proposals can be submitted via the study website (http://www.bristol.ac.uk/alspac/). We want to acknowledge the participants and investigators of the FinnGen study. Data is available from https://www.finngen.fi/en/access_results.
