# Supplementary material for: Assessing the causal effects of environmental tobacco smoke exposure: a meta-analytic Mendelian randomization study
Source: Nicotine Tob Res. 2026 Feb 25;28(8):1293–303. doi: 10.1093/ntr/ntag047 (PMC13389530; doi:10.1093/ntr/ntag047)
Supplement: Supplementary_Material_ntag047 [file supplementary_material_ntag047.zip › PS_Supplementary_Figure_S1_rv1_MM_bw_ntag047.docx]

**Supplementary Figure S1: STROBE-MR Flow Diagrams for each MR estimation approach and outcome.**

|  | Lung cancer | Chronic obstructive pulmonary disease (COPD) | Depression | Stroke | Coronary heart **disease (CHD)** | Hypertension |
| --- | --- | --- | --- | --- | --- | --- |
| Maternal smoking on index individual’s outcome | Lifetime smoking GWAS (N = 462 690; SNPs = 9,851,867)  Maternal smoking GWAS (N = 397,732; SNPs = 9,851,867)  120 SNPs after harmonising, clumping (r^2^ = 0.001 and MB = 10), and a p < 5 x 10^-8^ threshold.  Lung cancer GWAS (20,359 cases and 810,746 controls) | Lifetime smoking GWAS (N = 462 690; SNPs = 9,851,867)  Maternal smoking GWAS (N = 397,732; SNPs = 9,851,867)  100 SNPs after harmonising, clumping (r^2^ = 0.001 and MB = 10), and a p < 5 x 10^-8^ threshold.  COPD GWAS (58,559 cases, and 937,358 controls) | Lifetime smoking GWAS (N = 462 690; SNPs = 9,851,867)  Maternal smoking GWAS (N = 397,732; SNPs = 9,851,867)  124 SNPs after harmonising, clumping (r^2^ = 0.001 and MB = 10), and a p < 5 x 10^-8^ threshold.  Depression GWAS (170,756 cases and 329,443 controls) | Lifetime smoking GWAS (N = 462 690; SNPs = 9,851,867)  Maternal smoking GWAS (N = 397,732; SNPs = 9,851,867)  129 SNPs after harmonising, clumping (r^2^ = 0.001 and MB = 10), and a p < 5 x 10^-8^ threshold.  Stroke GWAS (1,234,808 cases and 1,308,460 controls) | Lifetime smoking GWAS (N = 462 690; SNPs = 9,851,867)  Maternal smoking GWAS (N = 397,732; SNPs = 9,851,867)  128 SNPs after harmonising, clumping (r^2^ = 0.001 and MB = 10), and a p < 5 x 10^-8^ threshold.  CHD GWAS (239,785 cases and 1,355,114 controls) | Lifetime smoking GWAS (N = 462 690; SNPs = 9,851,867)  Maternal smoking GWAS (N = 397,732; SNPs = 9,851,867)  128 SNPs after harmonising, clumping (r^2^ = 0.001 and MB = 10), and a p < 5 x 10^-8^ threshold.  Hypertension GWAS (242,724 cases, and 649,418 controls) |
| Maternal smoking on paternal outcomes | Paternal smoking GWAS (N = 5,766; SNPs = 9,425,631)  Maternal smoking GWAS (N = 397,732; SNPs = 9,851,867)  19 SNPs after harmonising, clumping (r^2^ = 0.001 and MB = 10), and a p < 5 x 10^-8^ threshold.  Paternal Lung cancer GWAS (37,443 cases 401,624 controls) | Paternal smoking GWAS (N = 5,766; SNPs = 9,425,631)  Maternal smoking GWAS (N = 397,732; SNPs = 9,851,867)  19 SNPs after harmonising, clumping (r^2^ = 0.001 and MB = 10), and a p < 5 x 10^-8^ threshold.  Paternal COPD GWAS (46,263 cases, 356,126 controls) | Paternal smoking GWAS (N = 5,766; SNPs = 9,425,631)  Maternal smoking GWAS (N = 397,732; SNPs = 9,851,867)  19 SNPs after harmonising, clumping (r^2^ = 0.001 and MB = 10), and a p < 5 x 10^-8^ threshold.  Paternal Depression GWAS (15,430 cases, 384,068 controls) | Paternal smoking GWAS (N = 5,766; SNPs = 9,425,631)  Maternal smoking GWAS (N = 397,732; SNPs = 9,851,867)  19 SNPs after harmonising, clumping (r^2^ = 0.001 and MB = 10), and a p < 5 x 10^-8^ threshold.  Paternal Stroke GWAS (62,810 cases, 339,806 cases) | Paternal smoking GWAS (N = 5,766; SNPs = 9,425,631)  Maternal smoking GWAS (N = 397,732; SNPs = 9,851,867)  19 SNPs after harmonising, clumping (r^2^ = 0.001 and MB = 10), and a p < 5 x 10^-8^ threshold.  Paternal Heart disease GWAS (133,320 cases and 274,237 controls) | Paternal smoking GWAS (N = 5,766; SNPs = 9,425,631)  Maternal smoking GWAS (N = 397,732; SNPs = 9,851,867)  19 SNPs after harmonising, clumping (r^2^ = 0.001 and MB = 10), and a p < 5 x 10^-8^ threshold.  Paternal Hypertension GWAS (91,242 cases and 311,657 controls) |
| Paternal smoking on maternal outcomes | Paternal smoking GWAS (N = 5,766; SNPs = 9,425,631)  Maternal smoking GWAS (N = 397,732; SNPs = 9,851,867)  19 SNPs after harmonising, clumping (r^2^ = 0.001 and MB = 10), and a p < 5 x 10^-8^ threshold.  Maternal Lung cancer GWAS (17,566 cases, 405,692 controls) | Paternal smoking GWAS (N = 5,766; SNPs = 9,425,631)  Maternal smoking GWAS (N = 397,732; SNPs = 9,851,867)  19 SNPs after harmonising, clumping (r^2^ = 0.001 and MB = 10), and a p < 5 x 10^-8^ threshold.  Maternal COPD GWAS (25,314 cases, 398,378 controls) | Paternal smoking GWAS (N = 5,766; SNPs = 9,425,631)  Maternal smoking GWAS (N = 397,732; SNPs = 9,851,867)  19 SNPs after harmonising, clumping (r^2^ = 0.001 and MB = 10), and a p < 5 x 10^-8^ threshold.  Maternal Depression GWAS (28,351 cases, 394,866 controls) | Paternal smoking GWAS (N = 5,766; SNPs = 9,425,631)  Maternal smoking GWAS (N = 397,732; SNPs = 9,851,867)  19 SNPs after harmonising, clumping (r^2^ = 0.001 and MB = 10), and a p < 5 x 10^-8^ threshold.  Maternal Stroke GWAS (60,880 cases, 364,097 cases) | Paternal smoking GWAS (N = 5,766; SNPs = 9,425,631)  Maternal smoking GWAS (N = 397,732; SNPs = 9,851,867)  19 SNPs after harmonising, clumping (r^2^ = 0.001 and MB = 10), and a p < 5 x 10^-8^ threshold.  Maternal Heart disease GWAS (85,620 cases and 340,620 controls) | Paternal smoking GWAS (N = 5,766; SNPs = 9,425,631)  Maternal smoking GWAS (N = 397,732; SNPs = 9,851,867)  19 SNPs after harmonising, clumping (r^2^ = 0.001 and MB = 10), and a p < 5 x 10^-8^ threshold.  Maternal Hypertension GWAS (130,948 cases and 426,391 controls) |
| Paternal smoking on index individual’s outcome | Paternal smoking GWAS (N = 5,766; SNPs = 9,425,631)  Lifetime smoking GWAS (N = 462 690; SNPs = 9,851,867)  116 SNPs after harmonising, clumping (r^2^ = 0.001 and MB = 10), and a p < 5 x 10^-8^ threshold.  Lung cancer GWAS (20,359 cases and 810,746 controls) | Paternal smoking GWAS (N = 5,766; SNPs = 9,425,631)  Lifetime smoking GWAS (N = 462 690; SNPs = 9,851,867)  100 SNPs after harmonising, clumping (r^2^ = 0.001 and MB = 10), and a p < 5 x 10^-8^ threshold.  COPD GWAS (58,559 cases, and 937,358 controls) | Paternal smoking GWAS (N = 5,766; SNPs = 9,425,631)  Lifetime smoking GWAS (N = 462 690; SNPs = 9,851,867)  122 SNPs after harmonising, clumping (r^2^ = 0.001 and MB = 10), and a p < 5 x 10^-8^ threshold.  Depression GWAS (170,756 cases and 329,443 controls) | Paternal smoking GWAS (N = 5,766; SNPs = 9,425,631)  Lifetime smoking GWAS (N = 462 690; SNPs = 9,851,867)  127 SNPs after harmonising, clumping (r^2^ = 0.001 and MB = 10), and a p < 5 x 10^-8^ threshold.  Stroke GWAS (1,234,808 cases and 1,308,460 controls) | Paternal smoking GWAS (N = 5,766; SNPs = 9,425,631)  Lifetime smoking GWAS (N = 462 690; SNPs = 9,851,867)  127 SNPs after harmonising, clumping (r^2^ = 0.001 and MB = 10), and a p < 5 x 10^-8^ threshold.  CHD GWAS (239,785 cases and 1,355,114 controls) | Paternal smoking GWAS (N = 5,766; SNPs = 9,425,631)  Lifetime smoking GWAS (N = 462 690; SNPs = 9,851,867)  127 SNPs after harmonising, clumping (r^2^ = 0.001 and MB = 10), and a p < 5 x 10^-8^ threshold.  Hypertension GWAS (242,724 cases, and 649,418 controls) |
| index individual’s smoking on index individual’s outcome | Lifetime smoking GWAS (N = 462 690; SNPs = 9,851,867)  113 SNPs after harmonising, clumping (r^2^ = 0.001 and MB = 10), and a p < 5 x 10^-8^ threshold.  Lung cancer GWAS (20,359 cases and 810,746controls) | Lifetime smoking GWAS (N = 462 690; SNPs = 9,851,867)  95 SNPs after harmonising, clumping (r^2^ = 0.001 and MB = 10), and a p < 5 x 10^-8^ threshold.  COPD GWAS (58,559 cases, and 937,358 controls) | Lifetime smoking GWAS (N = 462 690; SNPs = 9,851,867)  116 SNPs after harmonising, clumping (r^2^ = 0.001 and MB = 10), and a p < 5 x 10^-8^ threshold.  Depression GWAS (170,756 cases and 329,443 controls) | Lifetime smoking GWAS (N = 462 690; SNPs = 9,851,867)  120 SNPs after harmonising, clumping (r^2^ = 0.001 and MB = 10), and a p < 5 x 10^-8^ threshold.  Stroke GWAS (1,234,808 cases and 1,308,460 controls) | Lifetime smoking GWAS (N = 462 690; SNPs = 9,851,867)  120 SNPs after harmonising, clumping (r^2^ = 0.001 and MB = 10), and a p < 5 x 10^-8^ threshold.  CHD GWAS (239,785 cases and 1,355,114 controls) | Lifetime smoking GWAS (N = 462 690; SNPs = 9,851,867)  120 SNPs after harmonising, clumping (r^2^ = 0.001 and MB = 10), and a p < 5 x 10^-8^ threshold.  Hypertension GWAS (242,724 cases, and 649,418 controls) |

**Alt Text:** This study presents a series of forest plots showing the number of individuals and genetic variants included in each analysis.

**References to GWAS mentioned in this figure:**

Exposure GWAS:

Lifetime smoking GWAS: https://www.ncbi.nlm.nih.gov/pmc/articles/PMC7610182/

Maternal smoking GWAS: https://gwas.mrcieu.ac.uk/files/ukb-b-17685/ukb-b-17685_report.html

Paternal Smoking GWAS: for SNP selection <https://bmcresnotes.biomedcentral.com/articles/10.1186/s13104-023-06438-4>, and the GWAS used to derive estimates is described in Supplementary Methods.

Index individual outcome GWAS:

Lung cancer GWAS were a combination of: <https://www.ncbi.nlm.nih.gov/pmc/articles/PMC4074058/>, doi.org/10.5523/bris.aed0u12w0ede20olb0m77p4b9 and <https://r10.risteys.finngen.fi/endpoints/C3_BRONCHUS_LUNG_EXALLC>

COPD GWAS: http://results.globalbiobankmeta.org/pheno/COPD

Depression GWAS were a combination of: <https://www.ncbi.nlm.nih.gov/pmc/articles/PMC6522363/> and <https://r10.risteys.finngen.fi/endpoints/F5_DEPRESSIO>

Stroke GWAS: <https://www.ncbi.nlm.nih.gov/pmc/articles/PMC9524349/>

Coronary heart disease GWAS were a combination of: <https://www.ncbi.nlm.nih.gov/pmc/articles/PMC9729111/> and <https://r10.risteys.finngen.fi/endpoints/I9_CHD>

Hypertension GWAS were a combination of: <https://gwas.mrcieu.ac.uk/files/ukb-b-14057/ukb-b-14057_report.html> and <https://r10.risteys.finngen.fi/endpoints/I9_HYPTENS>

Maternal outcome GWAS:

Maternal Lung cancer GWAS: https://gwas.mrcieu.ac.uk/files/ukb-b-20176/ukb-b-20176_report.html

Maternal COPD GWAS: https://gwas.mrcieu.ac.uk/files/ukb-b-12018/ukb-b-12018_report.html

Maternal Depression GWAS: https://gwas.mrcieu.ac.uk/files/ukb-b-10807/ukb-b-10807_report.html

Maternal Stroke GWAS: https://gwas.mrcieu.ac.uk/files/ukb-b-4024/ukb-b-4024_report.html

Maternal Heart disease GWAS: https://gwas.mrcieu.ac.uk/files/ukb-b-12477/ukb-b-12477_report.html

Maternal Hypertension GWAS: https://gwas.mrcieu.ac.uk/files/ukb-b-18167/ukb-b-18167_report.html

Paternal outcome GWAS:

Paternal Lung cancer GWAS: https://gwas.mrcieu.ac.uk/files/ukb-b-14521/ukb-b-14521_report.html

Paternal COPD GWAS: https://gwas.mrcieu.ac.uk/files/ukb-b-9127/ukb-b-9127_report.html

Paternal Depression GWAS: https://gwas.mrcieu.ac.uk/files/ukb-b-5942/ukb-b-5942_report.html

Paternal Stroke GWAS: https://gwas.mrcieu.ac.uk/files/ukb-b-12777/ukb-b-12777_report.html

Paternal Heart disease GWAS: https://gwas.mrcieu.ac.uk/files/ukb-b-18408/ukb-b-18408_report.html

Paternal Hypertension GWAS: https://gwas.mrcieu.ac.uk/files/ukb-b-19456/ukb-b-19456_report.html
