# Supplementary material for: Assessing the causal effects of environmental tobacco smoke exposure: a meta-analytic Mendelian randomization study
Source: Nicotine Tob Res. 2026 Feb 25;28(8):1293–303. doi: 10.1093/ntr/ntag047 (PMC13389530; doi:10.1093/ntr/ntag047)
Supplement: Supplementary_Material_ntag047 [file supplementary_material_ntag047.zip › PS_Supplementary_Figure_S2_MM_bw_ntag047.docx]

**Supplementary Figure S2: Leave-one-out plots for the primary, common effects, meta-analysis.**

| 1. **lung cancer** | 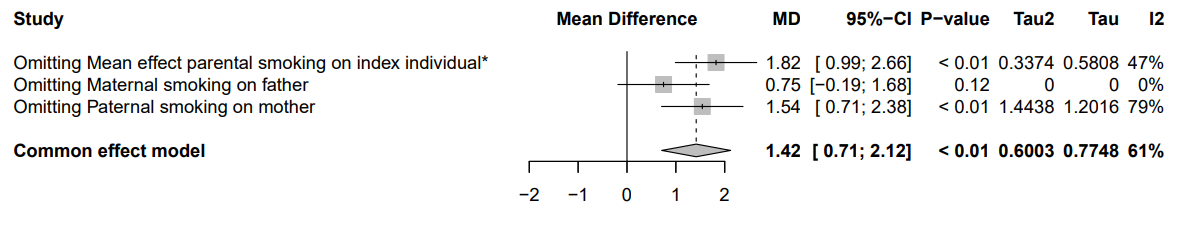 |
| --- | --- |
| 1. COPD | 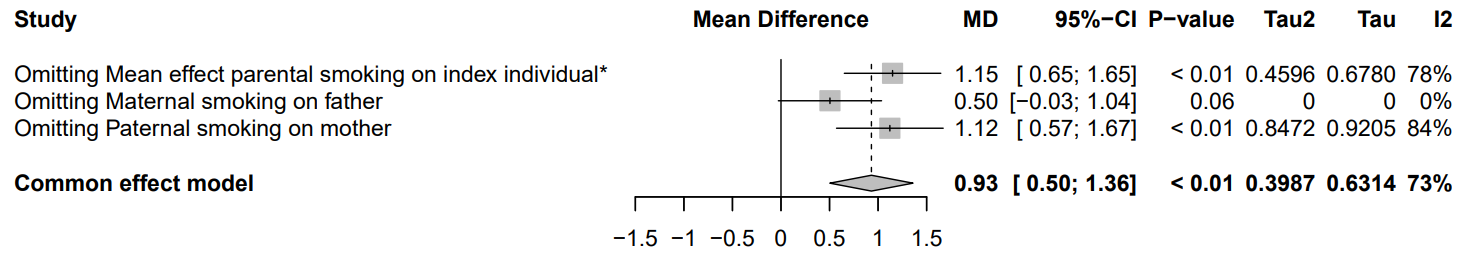 |
| 1. H**eart disease** | 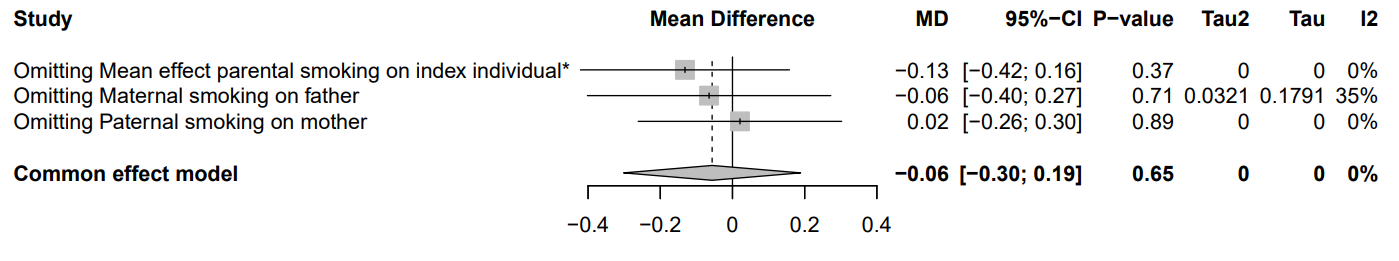 |
| 1. S**troke** | 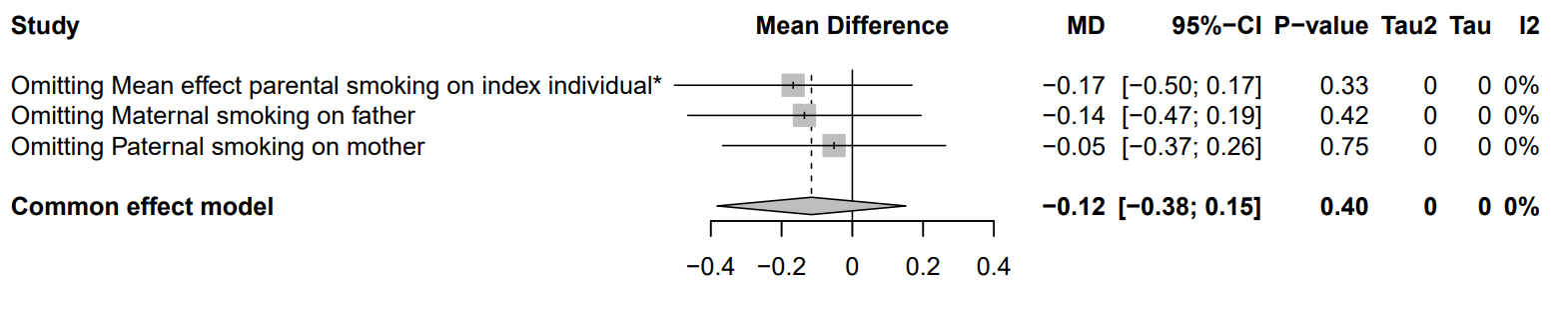 |
| 1. **Hypertension** | 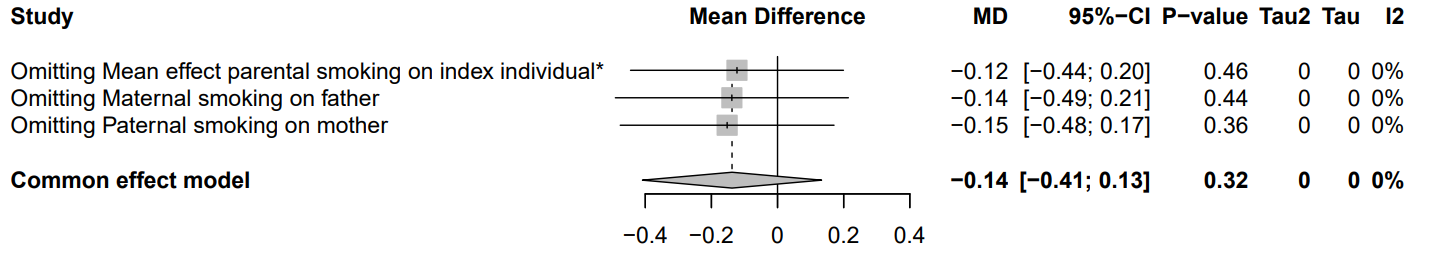 |
| 1. **Depression** | **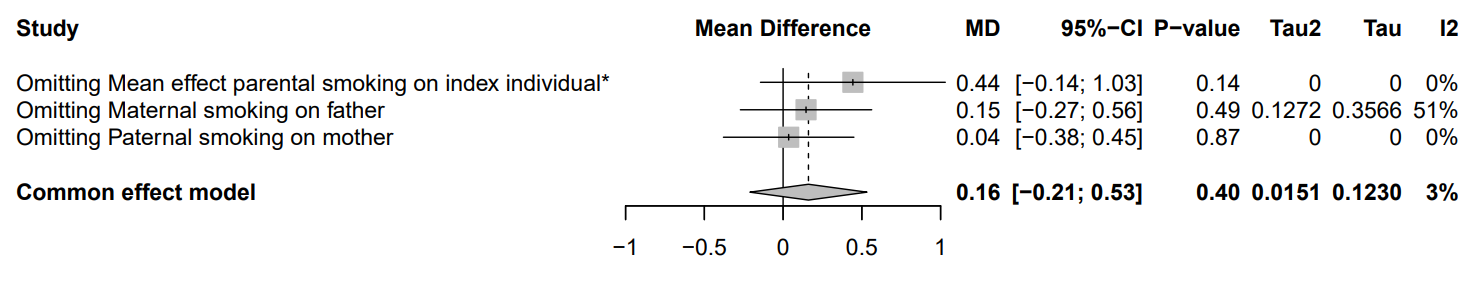** |

* We included the average of the estimates of maternal and paternal smoking on the index individual in the meta-analysis because these estimates are not independent.

**Alt Text:** This figure presents six leave-one-out plots for the meta-analysis.
