# Supplementary material for: Assessing the causal effects of environmental tobacco smoke exposure: a meta-analytic Mendelian randomization study
Source: Nicotine Tob Res. 2026 Feb 25;28(8):1293–303. doi: 10.1093/ntr/ntag047 (PMC13389530; doi:10.1093/ntr/ntag047)
Supplement: Supplementary_Material_ntag047 [file supplementary_material_ntag047.zip › PS_Supplementary_Figure_S3_ntag047.docx]

Supplementary Figure S3: Directed Acyclic Graphs (DAGs) illustrating how this recall bias can produce bias in our MR estimates.


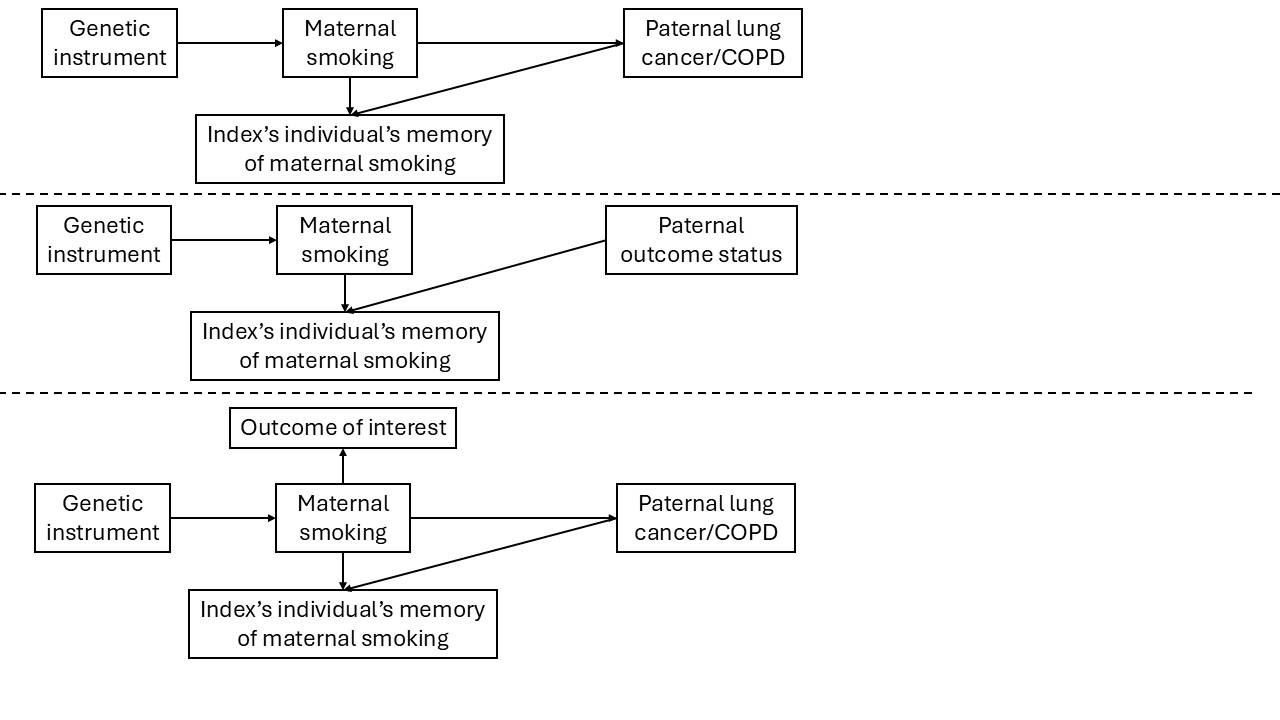


**Alt Text:** In the top DAG the association between the genetic instrument and the index individual’s memory of maternal smoking acts through both the direct effect of maternal smoking on the index individual’s memory of maternal smoking and the path mediated by the paternal lung cancer/COPD status (recall bias). Thus, when there is a causal effect, recall bias can impact on the size of MR estimates through this indirect effect. In the middle DAG there is no causal effect of smoking on the paternal outcome status. Since there is no path between the between maternal smoking and the individual’s memory of maternal smoking mediated by the paternal outcome status, outcome-related differences in the participant’s memory cannot impact on MR estimates when the null hypothesis is true. This means that recall bias cannot create false positive associations (assuming the core MR assumptions are otherwise valid). The bottom DAG shows that the presence of recall bias for lung cancer/COPD does not induce bias for other outcomes which do not impact on the participant’s memory of maternal smoking. The middle and bottom DAGs thus illustrate why extreme associations are not observed for potentially null and/or less recall bias prone (non-respiratory and/or index individual) outcomes.
