# Supplementary material for: Assessing the causal effects of environmental tobacco smoke exposure: a meta-analytic Mendelian randomization study
Source: Nicotine Tob Res. 2026 Feb 25;28(8):1293–303. doi: 10.1093/ntr/ntag047 (PMC13389530; doi:10.1093/ntr/ntag047)
Supplement: Supplementary_Material_ntag047 [file supplementary_material_ntag047.zip › PS_Supplementary_Figure_S4_ntag047.docx]

| **A) Lung cancer** | 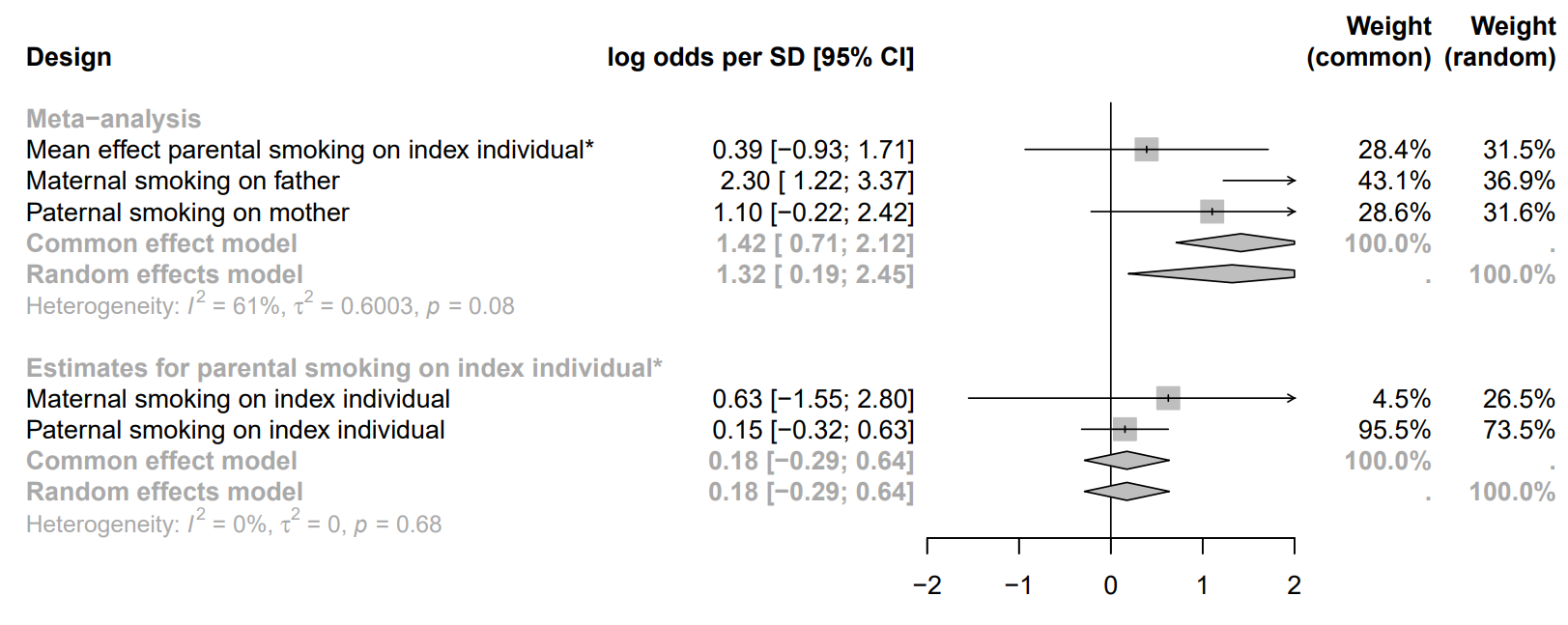 | B) COPD | 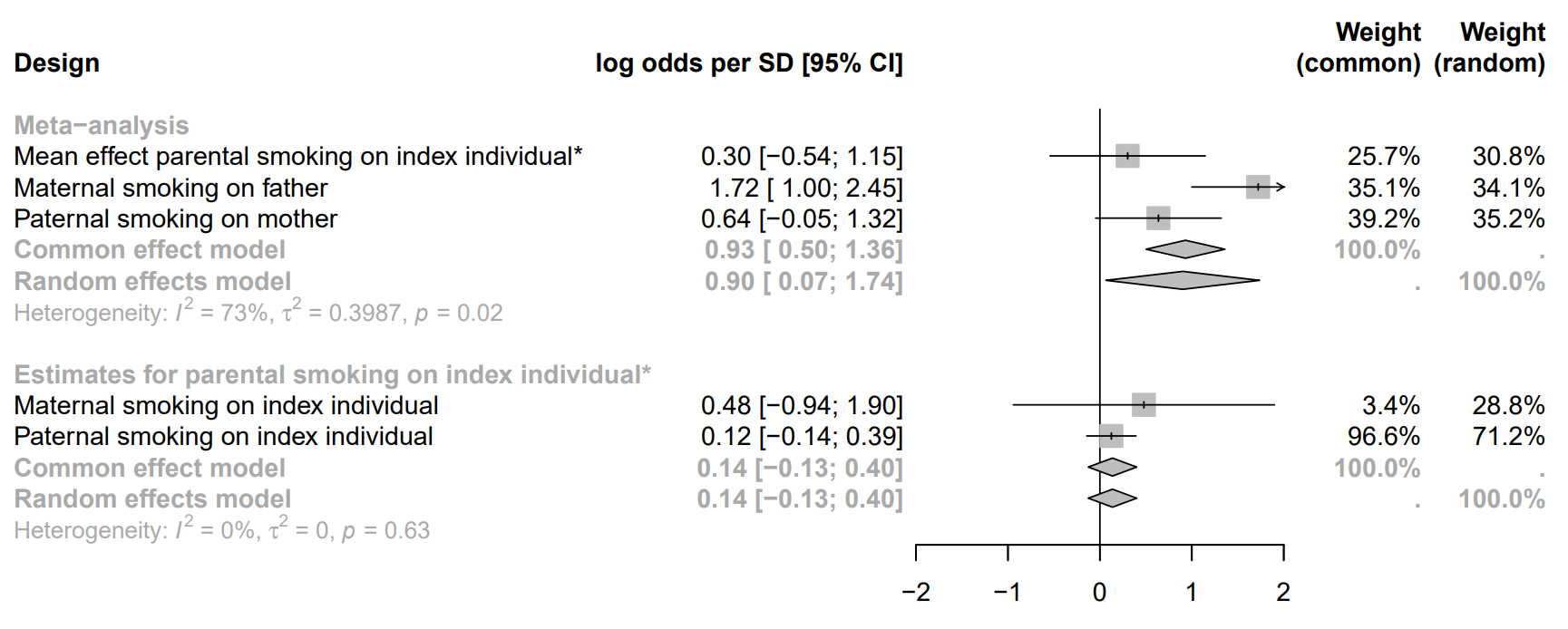 |
| --- | --- | --- | --- |
| **C) Coronary heart disease** | 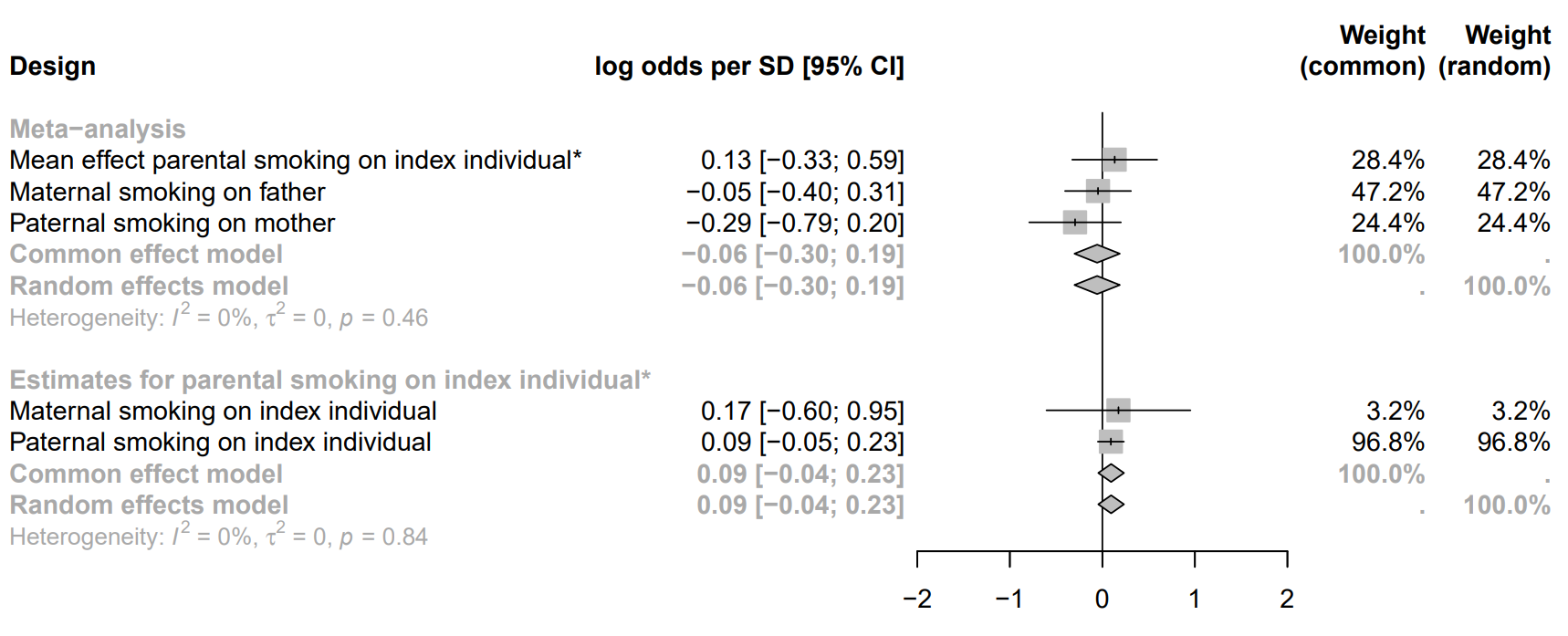 | **D) Depression** | 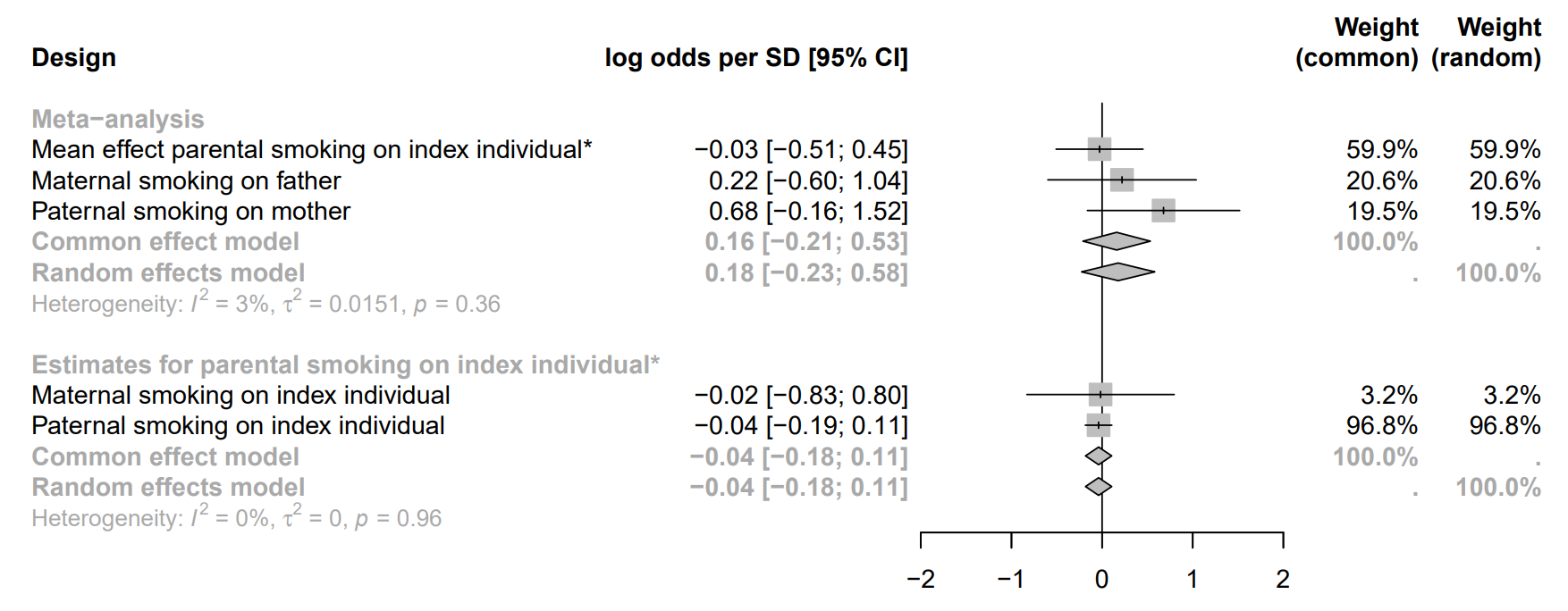 |
| **E) Hypertension** | 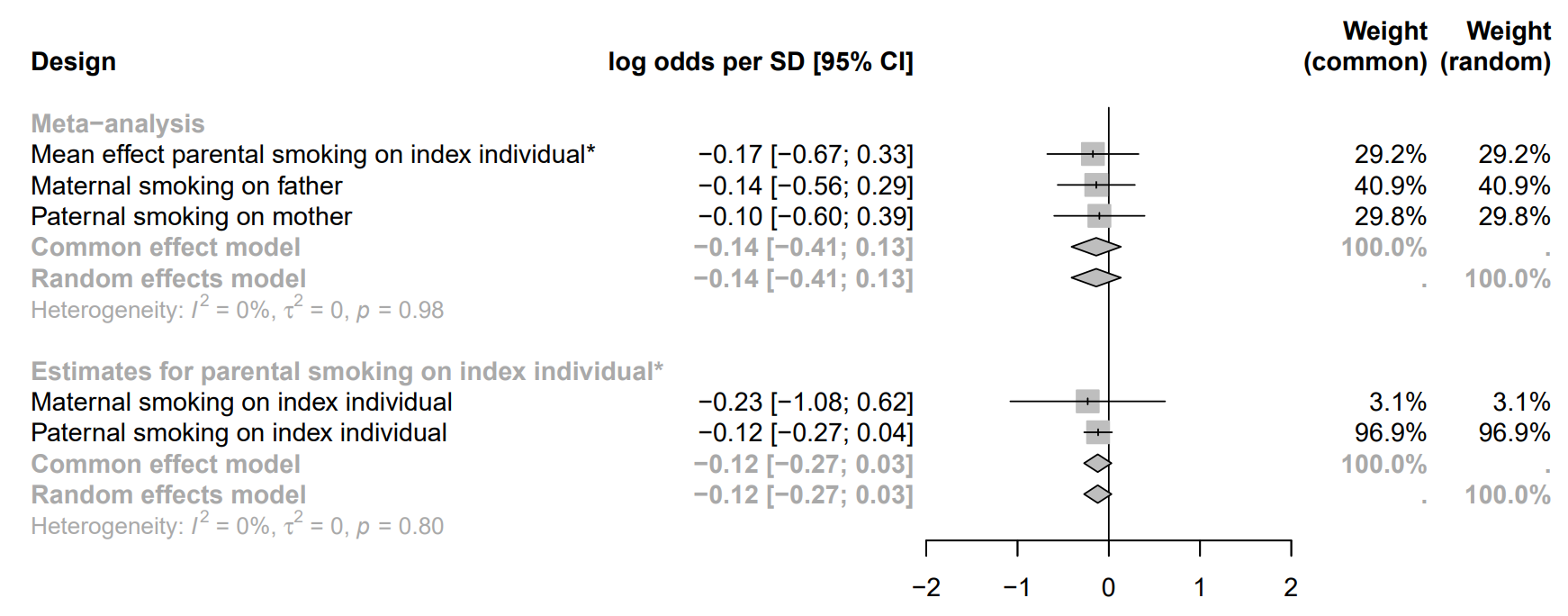 | **F) Stroke** | 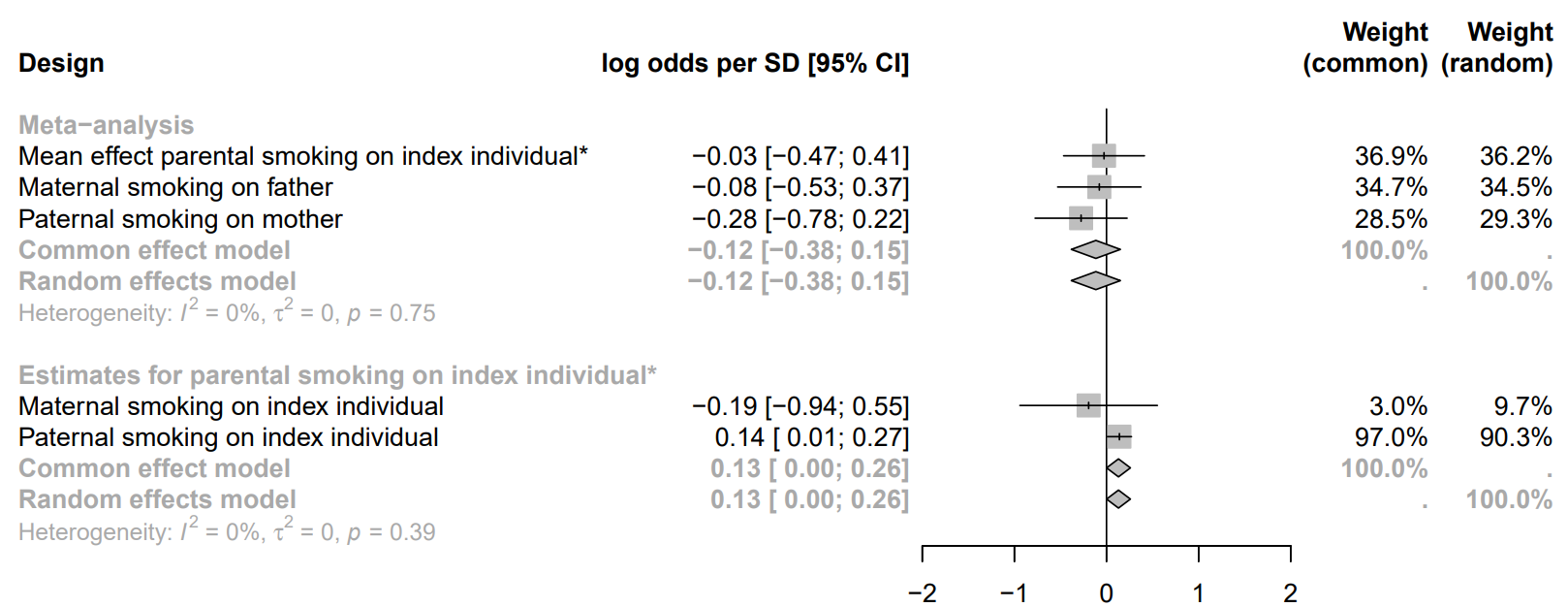 |

**Alt Text:** This figure presents six forest plots illustrating the results of the meta-analysis of the four Mendelian Randomization designs for the six outcomes.
