# Supplementary material for: Assessing the causal effects of environmental tobacco smoke exposure: a meta-analytic Mendelian randomization study
Source: Nicotine Tob Res. 2026 Feb 25;28(8):1293–303. doi: 10.1093/ntr/ntag047 (PMC13389530; doi:10.1093/ntr/ntag047)
Supplement: Supplementary_Material_ntag047 [file supplementary_material_ntag047.zip › PS_Supplementary_Methods_rv1_MM_bw_ntag047.docx]

**Supplementary Methods**

Assumptions of MR

MR is an application of instrumental variable (IV) analysis using genetic variants as instruments. Univariable IV makes three assumptions: 1) the variant is robustly associated with the exposure (relevance), 2) there are no variant-outcome confounders (independence), 3) the variant causes the outcome only through the exposure (exclusion restriction). In a multivariable setting, these assumptions are slightly modified: 1) the variant is robustly associated with each exposure conditional on the other exposure(s), 2) conditional on the exposures, there is no instrument-variant outcome confounding, and 3) that the instrument causes the outcome only through the exposures.

For the point estimate to be interpreted, IV analyses make a fourth assumption, which is typically monotonicity. This assumption allows the point estimate to be interpreted as the ‘complier’ average causal effect (i.e., the average effect among people whose genetically predicted smoking status is influenced by them inheriting the instrument) provided that there are no defiers (i.e., people who would be less likely to be smokers were they to inherit a variant which tends to increase the average liability of someone to smoke) (1). However, it is untestable in a summary data setting.

Two-sample MR requires that the data sources come from the same population for the effect estimate to be meaningful. In addition, if there is no sample overlap, then weak instrument bias will be towards the null in a univariable MR setting (2–4).

Because GWAS typically assume linearity, two-sample MR additionally assumes a linear effect. Traditional IV estimators, such as the Wald ratio used in two-sample MR will, however, still provide a valid estimate of the average causal effect in the presence of non-linearities (2).

Primer on summary data MVMR

Suppose that the following model holds:

β_Y_ = θβ_x_ + ε

where ε is a random normal error term with a mean of zero, θ the exposure-outcome effect, β_Y_ are the variant-outcome summary statistics, and β_x_ are the variant-exposure summary statistics.

The traditional IV Wald ratio for the ith variant is derived as follows:

β_Wald,i_ = β_Y,i_/β_X,i_

A conventional summary data MR analysis combines evidence from multiple variants by implementing an inverse variance weighted meta-analysis of the Wald ratios. This is equivalent to a inverse variance weighted intercept free regression of the variant-outcome associations on the variant-exposure associations. As such, it can be estimated using the following r code

lm(beta_y ~ beta_x + 0, weights = se_y^-2).

Where beta_y is a vector of variant-outcome associations, beta_x the variant-exposure associations, and se_y the standard errors for the variant-outcome associations.

MVMR is applied when there are multiple exposures which cause the outcome. The model is therefore extended so that:

β_Y_ = θ_x1_β_x1_ + θ_x2_β_x2_ + ε

As such, the MVMR model can be estimated using the following r code:

lm(beta_y ~ beta_x1 + beta_x2 + 0, weights = se_y^-2).

A link to the R code used in our analysis can be found at the end of the main text.

We additionally note that MR mediation methods are typically taken to estimate controlled direct effects (3,4)

Data sources

*UK Biobank (UKB).* The UKB is a population cohort study in the UK with around 500,000 participants, mostly of European ancestry. The study design, participants, and quality control (QC) methods have been described in full elsewhere (5). UKB received ethics approval from the North West Multi-Centre Research Ethics Committee (REC reference 11/NW/0382).

*Howard et al* (6)*:* This study (OpenGWAS ID: ieu-b-102) is a meta-analysis of two GWAS of depression scans with a combined sample of 500,199 individuals (170,756 cases and 329,443 controls). Specifically, the study includes 138,884 individuals from the Psychiatric Genetic Consortium (PGC), and 361,315 from the UKB. The PGC sample is itself a meta-analysis of around half a dozen smaller studies. The studies were overall around 50% female, and both samples were of broadly European ancestry individuals.

*Wang et al* (7) *and Burrows et al* (8)*:* These are two GWASs of lung cancer (OpenGWAS ID: ieu-a-966, ieu-b-4954, ieu-b-4955). The Wang et al. GWAS contains 11,348 cases and 15,861 controls from the International Lung Cancer Consortium (ILCCO). Specifically, they meta-analysed four European ancestry GWAS scans which all include male and female participants. Two of the included studies were from population-based samples, while two recruited cases and controls from a clinical setting. The Burrows et al. GWAS contains 2,671 cases and 372,016 controls from the UK Biobank described above. We meta-analysed SNP effects form these two GWAS scans using a common effect model.

*Mishra et al* (9)*:* The European-only GIGASTROKE GWAS is GWAS is a meta-analysis for STROKE with 73,652 cases and 1,234,808 from 44 studies. Where reported, the mean age of the recruited studies was generally in the participants’ mid-to-late 60s, with around 50% female. The included studies were a mixture of clinical and population-based settings.

*Aragam et al* (10): The European sub-sample of this GWAS meta-analysis of coronary artery disease (CAD) included studies are a mixture of clinical and population-based settings with both male and female participants. The study includes 181,522 European ancestry cases, and 984,168 European ancestry controls.

*Global Biobank Meta-Analysis*: The European sub-sample of the Global Biobank Meta-Analysis included 58,559 COPD cases and 937,358 controls European ancestry individuals recruited from: BioMe, BioVU, the Colorado Centre for Personalized Medicine, Estonia Biobank, FinnGen, Generation Scotland, HUNT, Lifelines, Mass General Brigham biobank, the Michigan Genomics Initiative, the UCLA Precision Health Biobank, and the UK Biobank.

*FinnGen*: FinnGen is a Finnish population Biobank, described in detail elsewhere (11,12). In brief, the FinnGen study is a large-scale genomics initiative that analysed over 500,000 Finnish biobank samples and correlated genetic variation with health data. the data used here come from the 10^th^ round of analyses which has genomic data in 230,310 females and 181,871 males (FinnGen. IDs: I9_HYPTENS, F5_DEPRESSIO, I9_CHD, and C3_BRONCHUS_LUNG_EXALLC).

*Avon Longitudinal Study of Parents and Children:* The Avon Longitudinal Study of Parents and Children (ALSPAC) is a birth cohort study of individuals born during 1991 and 1992 in the Avon aria which has been described in detail elsewhere (13,14). In brief, pregnant women resident in Avon, UK with expected dates of delivery between 1st April 1991 and 31st December 1992 were invited to take part in the study. 20,248 pregnancies were identified as being eligible and the initial number of pregnancies enrolled was 14,541. Of the initial pregnancies, there was a total of 14,676 foetuses, resulting in 14,062 live births and 13,988 children who were alive at 1 year of age. When the oldest children were approximately 7 years of age, an attempt was made to bolster the initial sample with eligible cases who had failed to join the study originally. As a result, when considering variables collected from the age of seven onwards (and potentially abstracted from obstetric notes) there are data from an additional 913 children being enrolled (456, 262 and 195 recruited during Phases II, III and IV respectively). The phases of enrolment are described in more detail in the cohort profile paper and its update (13,14). The total sample size for analyses using any data collected after the age of seven is therefore 15,447 pregnancies, resulting in 15,658 foetuses. Of these 14,901 children were alive at 1 year of age. We included 5,766 unrelated individuals of European descent form the study who had given consent for genotyping and had had paternal smoking phenotyped. Basic demographic information is presented in the table below:

| **Phenotype** | **Mean** | **SD** | **N missing*** |
| --- | --- | --- | --- |
| Father’s age at recruitment | 31.2 | 5.6 | 357 |
| Father’s smoking at offspring age 1 | 3.2 | 7.0 | 1087 |
| Father’s smoking at offspring age 8 | 2.5 | 6.3 | 1758 |
| **Phenotype** | **N** | **%** | **N missing** |
| Father smoked at either age 1 or age 8 | 1642 | 37.4 | 644 |
| Offspring smokers at age 16 | 1736 | 46.9 | 3412 |
| Offspring sex (Female) | 3268 | 48.9 | 0 |
| Father has a degree | 5081 | 77.9 | 162 |

* Total N = 6681, the number of unrelated individuals of European descent

Please note that the ALSPAC study website contains details of all the data that is available through a fully searchable data dictionary and variable search tool via the following webpage: http://www.bristol.ac.uk/alspac/researchers/our-data

Phenotyping

*UK Biobank:* Below we present the variable codes for the UK Biobank measures used.

Information on hair colour (UKB ID: 1747, OpenGWAS IDs: ukb-d-1747_5, ukb-d-1747_4, ukb-d-1747_3, ukb-d-1747_1, ukb-d-1747_2, ukb-d-1747_6) was ascertained through a questionnaire asked at baseline assessment. Participants were asked: "What best describes your natural hair colour? (If your hair colour is grey, the colour before you went grey)". There were 15,809 cases with black hair, 41,178 cases with blonde hair, 134,627 with dark brown hair, 147,560 with light brown hair, 16,615 with red hair, and 4,481 with another colour.

Illness of mother (UKB ID: 20110, OpenGWAS IDs: ukb-b-18167, ukb-b-10807, ukb-b-12018, ukb-b-20176, ukb-b-4024, ukb-b-12477) was ascertained through a questionnaire asked at baseline assessment. Participants were asked: "Has/did your mother ever suffer from? (You can select more than one answer)" and then presented with a list of 12 illnesses (prostate cancer, severe depression, Parkinson's disease, Alzheimer's disease/dementia, diabetes, high blood pressure, chronic bronchitis/emphysema, breast cancer, bowel cancer, lung cancer, stroke, heart disease). There were 60,880 cases of maternal stroke cases with 85,620 cases of maternal heart disease, 130,948 cases of maternal hypertension, 25,314 cases of maternal chronic obstructive pulmonary disease, 17,566 cases of maternal lung cancer, and 28,351 of maternal depression. These GWAS scans had total sample sizes ranging from 394,866 (depression) to 426,391 (hypertension).

Illness of father (UKB ID: 20107, OpenGWAS IDs: ukb-b-18408, ukb-b-19456, ukb-b-12777, ukb-b-9127, ukb-b-14521, ukb-b-5942) was ascertained through a questionnaire asked at baseline assessment. Participants were asked: "Has/did your father ever suffer from? (You can select more than one answer)" and then presented with a list of 12 illnesses (prostate cancer, severe depression, Parkinson's disease, Alzheimer's disease/dementia, diabetes, high blood pressure, chronic bronchitis/emphysema, breast cancer, bowel cancer, lung cancer, stroke, heart disease). There were 62,810 cases of paternal stroke cases with 133,320 cases of paternal heart disease, 91,242 cases of paternal hypertension, 46,263 cases of paternal chronic obstructive pulmonary disease, 37,443 cases of paternal lung cancer, and 15,430 of paternal depression. These GWAS had total sample sizes ranging from 399,499 (depression) to 407,557 (heart disease).

Maternal smoking status was measured (UKB ID: 1787, OpenGWAS ID: ukb-b-1768) was ascertained through a questionnaire asked at baseline assessment. Participants were asked: "Did your mother smoke regularly around the time when you were born?". There were 121,634 cases, and 276,098 controls.

Hypertension (UKB ID: 20002, OpenGWAS ID: ukb-b-14057) was ascertained through a questionnaire asked at baseline assessment. Participants were verbally asked, during an interview, what non-cancer illness they had. There were 119,731 cases, and 343,202 controls.

We additionally used information on paternal smoking (see Woolf et al (15,16)) and lifetime smoking (see Wooton et al (17)). These were derived from other phenotypes and have been described in the references given in parentheses.

*Howard et al*: This study did not provide a clear description of the measures used by the included simples. However, the descriptor papers of the two studies meta-analysed state that, the UKB participants were phenotyped based on “three depression-related phenotypes: broad depression, probable major depressive disorder (MDD), and International Classification of Diseases (ICD, version 9 or 10)-coded MDD” (18). The PGC sample used a mixture of self-report, medical records, and structured interviews to define cases (19).

*Wang et al and* Burrows *et al:* Three out of four of the studies in Wang et al used a clinical diagnosis of lung cancer, while one used a self-report measure. More details are available in the original publication (7). Burrows et al used ICD code defined cases.

*Mishra et al:* This study included studies that generally required clinical diagnosis as the case definition for stroke. More details are available in the original publication (9)*.*

*Aragam et al:* This study included studies that generally required clinical diagnosis as the case definition for CAD. More details are available in the original publication (10).

*Global Biobank Meta-analysis*: The included biobanks generally identified cases through medical record linkage.

*FinnGen:* This study defined cases according to medical records. More details are available in the original publication (11,20).

*ALSPAC*: Paternal smoking status (during the participants childhood) was measured by a questionnaire filled out by the participants’ mothers when the offspring were aged 1 and 8. Fathers who smoked at either time were coded as smokers. When information was missing for one time point, paternal smoking was assessed using the non-missing time point. Around 1000 participants were excluded for having no information on paternal smoking collected at either time point.

Genotyping

*UKB:* All UKB GWAS scans, including those from Burrows et al, and Wooton et al, were conducted using the MRC-IEU UKB GWAS pipeline (21). A full description of the pipeline methods can be found elsewhere (21). In brief, the summary statistics were created using a linear mixed model implemented in BOLT-LMM (22), adjusting for sex and SNP-chip. Links to the QQ and Manhattan plots for these GWASs can be found in Supplementary Figure S1.

Because they were also not publicly available, we additionally ran our own GWAS of maternal smoking, mother’s and father’s lung cancer and chronic obstructive pulmonary disease, using the MRC-IEU UKB GWAS pipeline but without adjusting for genotyping chip. To improve the computation efficiency of BOLT-LMM we additionally adjusted for the first 10 principle concepts of ancestry in our BOLT GWAS scans.

The UKB GWAS of paternal smoking were created using a GWAS by subtraction, which is described elsewhere (15,16).

*Howard et al*: Genotyping and QC information about the included samples are described in the original publications meta-analysis (18,19,23). In brief, Howard et al filtered genetic variants with an imputation accuracy less than 0.6 or a minor allele frequency less than 0.005. LDSC regression intercepts for each included study were then further used to control for genomic inflation before meta-analysing. The LDSC intercept for the GWAS summary statistics used is 1.015 implying the presence of a negligible amount of residual population structure.

*Wang et al:* Genotyping and QC information about the included samples are described in the original publication (7). In brief, they excluded individuals with extremely high or low heterozygosity, low all rates, who were of non-European ancestry, or who were first-degree relatives of another participant. The LDSC intercept for the GWAS is 1.09, implying the presence of a small amount of residual population structure. We, therefore, applied this as a genomic control correction factor to the GWAS summary statistics.

*Mishra et al:* Genotyping and QC information about the included samples are described in the original publication (9). Effects were estimated using an additive effect model, adjusting for age, sex, principal components of ancestry and when needed study spec covariates.

*Aragam et al:* Genotyping and QC information about the included samples are described the original publication (10). Population structure was addressed by adjust for ancestry, which for all except 1 cohort involved controlling for at least the first 4 principal components of ancestry as well as some study specific covariates.

*Global Biobank Meta-analysis*: Biobanks were asked to adjust for age, age^2, sex, age*sex, the first 20 principle components of ancestry, and any biobank specific covariates (24).

*FinnGen:* Genotyping used Illumina and Affymetrix arrays. GWAS models adjusted for age, sex, the first 10 genetic principal components, and genotyping batch. Further details about the GWAS methods, including QC procedure, can be found on the documentation on their website (25).

*ALSPAC*: Our GWAS of paternal smoking used index individual genotypes with the 1000 genomes used as a reference genotype for imputation. A standardised quality control had been applied to these variants, and is described at:

<https://proposals.epi.bristol.ac.uk/alspac_omics_data_catalogue.html>.

We additionally removed SNPs with a minor allele frequency less than 1% or fewer than 20 individuals per allele. Our GWAS controlled for the father’s age, offspring’s sex, and the first 20 principal components of ancestry. The genomic control Lambda for the resulting GWAS was 1.007. The QQ and Manhattan plots for the resulting GWAS are presented bellow (and imply minimal genomic inflation beyond expectation):


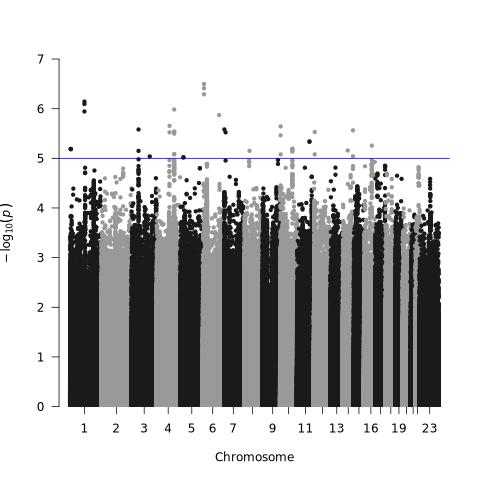

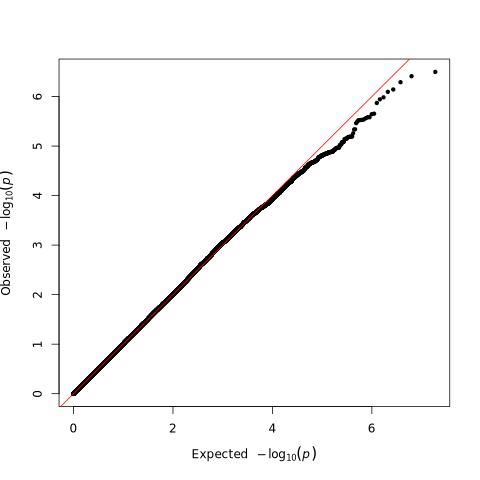


Further notes on smoking phenotypes used

Due to the importance of the smoking phenotypes to our study, we felt it would be useful to add some additional notes to the factual description of the measures used presented in the section on ‘Phenotyping’ above.

We used index individual lifetime smoking index because it is one of the better powered smoking phenotypes (17). Lifetime smoking index captures whether someone started smoking, the duration of their smoking, the heaviness of their smoking, and smoking cessation. Of these, smoking heaviness has the least pleiotropic genetic liability (26).

Maternal and paternal smoking instead measured smoking status. For mothers, this was at the index individual’s birth and was measured retrospectively through questionnaire given to the index individual. For fathers smoking status was if they smoked either when the child was aged 1 or 8. This was measured at time through maternal reporting. Smoking status will crudely capture smoking initiation (if someone never initiates they cannot be a smoker), duration and cessation (they have to have continued smoking from initiation to when the question is asked). Due to only using one or two time points and being a dichotomous variable, smoking status is a noisy measure of these dimensions of smoking. Because it is a binary indicator, it also cannot measure smoking heaviness. As such, the genetic risk score is a crude measure of the parental liability to being a smoker.

The index individual measure of smoking is therefore a broader (and more accurate) measure than the one used for parents. We believe that this should strengthen the design: since we are using offspring smoking to control for potential pleiotropic effect, using a broader phenotype should reduce the risk of a residual exclusion restriction violation.

Statistical methods for the MR analyses

The Wald ratio, defined by the variant-outcome association divided by the variant-exposure association, was the primary MR estimator in this study was the Wald ratio. BOLT-LLM produces estimates for binary phenotypes on the risk difference scale. To standardise effect estimates all BOLT-LMM GWAS scans were converted into the log odds ratio scale by dividing their effect estimates (and standard errors) by p(1-p), where p is the prevalence of the exposure.

The smoking GWAS scans, where necessary, had their effect estimates converted into standardised mean differences (SMD). We therefore converted maternal smoking from the log-odds scale to a SMD by dividing the estimates by $\left( \pi*3^{-0.5} \right)$ (27). We standardised the lifetime smoking summary statistics by dividing the beta and standard error of the summary statistics by 0.6940093 (one SD of lifetime smoking, which represents smoking 20 cigarettes a day for 15 years then stopping, or smoking 60 cigarettes a day for 13 years then stopping). This means that all MR estimates have units of the log odds of outcome per SD increase in exposure. In addition, because all GWAS scans used here assume linear models, our MR estimates also assume a linear effect.

In instances where there were multiple SNPs, we meta-analysed the SNP-specific Wald ratios. An inverse-variance weighted (IVW) meta-analysis will return the true effect if all the IV assumptions are valid.

We used the *TwoSampleMR* R package to harmonise the exposure and outcome data (28). Palindromic SNPs were only excluded if *TwoSampleMR* could not use the allele frequency to infer which strand was positive. Where possible, we imputed LD proxy variants with *TwoSampleMR* when SNPs were missing in the outcome dataset, using an r2 of 0.8 from the European subsample of the 1000 genomes project.

We additionally used three ‘pleiotropy robust’ methods. These are unbiased even if some of the instruments are invalid, but have reduced power. If we conceptualise IVW as an intercept free regression of the variant-outcome association on the variant-exposure association (29), then MR-Egger is the same model but allows for a non-zero intercept. MR-Egger requires that the variant-exposure effect size is independent of the size of any bias (such as a pleiotropic effect), called the INSIDE assumption. Additionally, MR-Egger assumes that there is no measurement error in the exposure GWAS (called the NOME assumption) (30,31). This assumption is relaxed by implementing MR-Egger using SIMEX regression (32). Simex regression can correct for regression dilution bias in regression estimates, which is equivalent to a violation of the NOME/no weak IV assumption in MR-Egger regression. Weighted mode assumes that the modal effect size is a valid estimate of the true effect size (i.e., zero modal pleiotropy or ZEMPA assumption), weighted median assumes that at least half of the SNPs are valid.

Finally, we also use four ‘weak instrument robust’ methods. Debias-IVW uses theoretically derived bias equations to try to anelasticity estimate and correct for bias in MR estimates (33). The generalised methods of moments (GMM) estimator minimises the sample moment conditions (34), and has similar weak-instrument robustness properties to the limited information maximum likelihood (LIML) estimator. MR Grapple uses a profile maximum-likelihood maximisation to address weak instrument bias, and can be thought of as a multivariable implementation of the widely used MR-RAPS (35). Qhet mitigates the effect of weak instrument bias by using an algorithm to minimise the heterogeneity between SNPs (36).

Note that while MR-GRAPPLE was introduced as a framework for conducting MR analyses, we were primarily interested in the estimator’s weak instrument robust properties and therefore implemented MR-GRAPPLE with the same genetic variants used for the other estimators. Although the MRBEE and CWBLS estimators have both been introduced as weak instrument robust estimators, we did not implement them here due to their similarity to debias IVW.

Bespoke risk of bias tool for MR studies

GRADE evaluations include an analysis of the risk of bias of the included studies. To our knowledge, there is no existing tool for assessing the risk of bias in MR approaches (or studies more generally). We therefore created a bespoke tool based on the recent systematic review of tools to assess risk of bias in MR studies by Spiga and colleagues (37). Ratings were made initially by BW and then ratified by SR.

Our bespoke risk of bias tool assesses the MR assumptions for each MR estimation approach. Our tool assessed risk of bias in 10 items structured into 8 domains: 1) the relevance assumption (i.e., that the genetic variants are strongly associated with the exposure), 2) the independence assumption (i.e., that there is no genetic variant-outcome confounding), 3) the exclusion restriction assumption (i.e., that there is no effect of the genetic variants on the outcome except via the exposure), 4) harmonisation of the SNP effects across samples, 5) that the samples were drawn from comparable populations, 6) clumping of the genetic instruments, 7) Winner’s Curse, 8) other risks of bias. Based on the ROB tool for clinical trials, we ranked studies as having a low, high, or unclear risk of bias in each domain (38). Studies with a low risk of bias in all domains were classed as low risk of bias. If a study was at high risk of bias for any domain, then it was at a high risk, otherwise, it was at an unclear risk of bias.

The first domain (relevance) included one item on the F-statistic of the variant-exposure association. Weak instrument bias is inversely proportional to the F-statistic of the variant-exposure association. By convention, an F-statistic of ten or greater is regarded as sufficient for minimal weak instrument bias, and we therefore used this threshold as the basis for assigning study risk of bias for the first item in this domain. Since all the ‘weak-instrument robust’ estimators we apply here use different approaches to relax the assumption of strong instruments, if these estimators provided consistent estimates, then we did not require downgrade to the study’s risk of bias even if a conditional F statistic was less than 10.

The second domain covers the independence assumption. A limitation of using population GWAS data is that resulting MR Estimates can be biased by from residual confounding, for example due to assortative mating, dynastic effects, or population structure (39). We evaluate risk of bias using had three items. A) If any of the exposure phenotypes had failed the hair colour negative control analyse. B) If the GWAS had applied a statistical method to control for population structure, e.g. by adjusting for principal components of ancestry, using a linear mixed model (such as BOLT-LMM), or applying genomic control (e.g. by dividing estimates by the LD Score Regression intercept (40)). In addition, if any GWAS adjusted for a potential collider then the study would be ranked as high or unclear risk of bias. C) The final item required that each study had fully adjusted for any potentially biasing paths presented in the DAGs in Figure 2 for the study to be ranked as low. Please note that estimates in the parental smoking on offspring outcome approaches could be impacted by assortative mating. Assortative mating is not represented in the DAGs for these designs because it is the reason for treating them as correlated in the meta-analyses (i.e., testing the hypothesis that either parent’s smoking causes offspring outcomes). From the perspective of hypothesis testing, this procedure should prevent an inflated false positive rate in the meta-analysis due to assortative mating in these designs. Assortative mating was therefore not evaluated for these two designs.

The third domain (exclusion restriction) domain contained one item. This item was on the use of standard sensitivity analysis in MR for the presence of pleiotropy. Horizontal pleiotropy occurs when a genetic variant is associated with two phenotypes for independent reasons. If horizontal pleiotropy is present, the exact pathway should be different for each variant. It has therefore been argued that, if it occurs, it should create heterogeneity in the MR estimates, and its presence can therefore be tested using a heterogeneity statistic. We required that the Cochrane Q statistic of the MR Wald ratios was not significant. If it was, then we additionally required homogeneity across the pleiotropy robust estimators for the study to be rated as low risk of bias. If these criteria were not met, the study was rated at high risk of bias for this item.

In the fourth domain (harmonisation) we required that the direction of effect estimates of all GWAS scans used in the study were aligned to be in the same direction, and that there was a sensible treatment of palindromic SNPs (e.g., excluding palindromes which could not be aligned), in order to be rated as low risk of bias.

The fifth domain tested the two-sample MR assumption that the samples are drawn from comparable populations. We required that samples were either drawn from the sample study (e.g., UKB), or have similar demographics.

Studies were ranked as low of bias if they clumped genetic variants with reasonable clumping parameters (e.g., r^2^ of 0.01 and KB of 1,000), or accounted for the correlation between genetic variants in a statistical model.

The ‘Other risk of bias’ domain includes an item on any other sources of bias, such as differential measurement error, selection bias, failure of the positive control for the outcome (i.e., that an individual’s smoking did not causes the outcome in that individual), or heterogeneity between the primary analysis and the analyses using UKB GWASs that did not adjust for genotyping chip.

The final domain assessed risk of bias due to Winner’s Curse. We required that studies either used a three-sample MR design (41), or a statistical correction for Winner’s Curse to be rated at low risk of bias.

Unlike Spiga and colleagues, we have not included sample overlap in our risk of bias tool (37). The effect of sample overlap is that weak instrument bias can shift univariable MR estimates in either direction, rather than to necessarily attenuate results towards the null. However, MVMR analyses can be biased by weak instrument bias in either direction even with no sample overlap. In addition, conducting the analysis with non-overlapping samples (e.g., by splitting the samples in half) would reduce the size of the exposure/outcome GWAS, and greatly increase the magnitude of weak instrument bias or reduce power. Thus, in this study we opted for the approach that minimised the overall magnitude of weak instrument bias while maximising power, and did not use sample overlap as a consideration when evaluating bias. For clarity, we note that since all outcome estimates include data from the UK Biobank, only estimates of paternal smoking (which are derived from ALSPAC) on offspring outcomes do not have sample overlap.

PRISMA checklist items not included

Because our study is a hybrid of a meta-analysis an MR study, certain items in the PRISMA and STROBE-MR guidelines are less appropriate. Specifically, the “eligibility criteria”, “information sources”, “search strategy”, “selection process”, “data collection”, “data items”, and “reporting bias assessment” from the methods section of the PRISMA checklist were not included because they are relevant to a systematic review. The “Reporting bias”, “Study selection”, and “Study characteristics” from the results of the PRISMA checklist were not included because these are subsumed in the description of the STROBE-MR.

Software and pre-registration

MR analyses in this paper were run using the TwoSampleMR, MVMRmode, MendelianRandomisation, MVMR, SIMEX, mr.divw, and ‘meta’ R packages in Rv4.1.1 (28,32,33,42–46). Much of the GWAS data was extracted from the MRC-IEU OpenGWAS platform (47). This study was not pre-registered.

**Evaluation of alternative summary data MR estimation approaches for assessing the effects of ETS**

Alternative approaches for estimating the effects of parental smoking on children

Here we have run a separate MVRM model of each parent conditional on the offspring’s genetic liability to smoke. However, people tend to partner with someone who has a similar smoking status to themselves, and smoking is known to be socially transmittable. Thus, as we note in the main text and above, the estimates for maternal and paternal smoking on offspring outcomes might not be independent of each other. Here we chose to account for this by averaging the estimates from both models. Since our primary aim is to test the joint null hypothesis that someone else’s smoking cannot increase disease risk in another individual, this procedure will not result in false positive associations, but it does mean that the estimates of maternal or paternal smoking cannot be interpreted as the unique effect of that parent.

An alternative approach would be to include both parent’s and offspring liability to smoke a single model. This would estimate the effect of each parent’s smoking independent of the other parent and the offspring. Given the low F statistics in the single-parent and child models, we did not adopt this strategy because of the risk that adding an additional genetically correlated exposure phenotype would simply result in highly unreliable estimates.

Using variants associated with self reported exposure to environmental tobacco smoke

We have not directly considered ‘third-hand smoking’, exposure to cigarette smoke in the more general environment which lacks a specific source. In an earlier version of this manuscript, we had two approaches which used genetic variants associated with self reported exposure to ETS. On reflection, we decide that this approach is likely unreliable. We are highlighting it here, both for the sake of transparency, and also to caution other researchers who might consider a similar approach.

Although many aspects of the environment are heritable, the causal mechanism between genetic variants and genuine environmental risks is often complex and not understood. Our prior research with caffeine and drug use phenotypes established that when the mechanism linking the variants to the exposure is not understood, MR studies using downstream phenotypes are at risk of misidentification and bias (48,49). This has now been demonstrated as a more general issue when instrumenting distal phenotypes (50). In the case of ETS, the most plausible mechanism is that the variants act through first-hand smoking.

Two plausible methods for exploring this hypothesis are to a) compare the effects on lung cancer (OpenGWAS ID: ieu-a-966) of variants selected as instruments for ETS to those known to instrument first-hand smoking. If a nearly identical estimate for the effect of first-hand smoking on lung cancer is observed when using a biologically validated first-hand smoking variant and the ETS variants, this indicates that the ETS variants are actually acting through first hand smoking. b) the look at the attenuation in effects after adjustment for first hand smoking. If the effect does not exist after adjusting for first-hand smoking, this again implies that what is observed is in fact an effect of first-hand smoking.

For the first analysis we used rs16969968 which has been biologically implicated with nicotine metabolism, and therefore smoking heaviness (51). This variant has a beta of 1.690 (se = 0.139) for the effect of smoking heaviness (OpenGWAS ID: ieu-b-142) on lung cancer (OpenGWAS ID: ieu-a-966). The results of the two exposures can be found in the two tables printed bellow:

| **SNPs associated with ETS exposure not at home (OpenGWAS ID: ukb-b-** **6244)** | **p < 5 x 10^-8^** | **p < 5 x 10^-7^** |
| --- | --- | --- |
| N SNPs | 6 | 16 |
| ETS UVMR beta (se) of lung cancer on ETS exposure not at home | 0.633 (2.092) | 1.428 (1.054) |
| ETS MVMR beta (se) of lung cancer on ETS exposure not at home conditional on smoking heaviness | -1.193 (1.651) | -0.299 (1.073) |
| ETS UVMR beta (se) of lung cancer on smoking heaviness | 1.808 (0.719) | 1.537 (0.463) |

| **SNPs associated with ETS exposure at home**  **(OpenGWAS ID: ukb-a-19)** | **p < 5 x 10^-8^** | **p < 5 x 10^-7^** |
| --- | --- | --- |
| N SNPs | 0 | 1 |
| ETS UVMR beta (se) of lung cancer on ETS exposure at home | NA | 2.436 (3.908) |
| ETS MVMR beta (se) of lung cancer on ETS exposure at home conditional on smoking heaviness | NA | NA |
| ETS UVMR beta (se) of lung cancer on smoking heaviness | NA | 31.403 (50.377) |

The first table shows the results for self reported ETS exposure outside of the home. Consistent with our hypothesis, the univariable MR estimate for the effect of smoking on lung cancer using SNPs associated with ETS outside of the home were very similar to those estimated using rs16969968. Secondly, after adjusting for smoking heaviness, the effect of ETS exposure at home was attenuated. Both of these results therefore indicate that the variants associated with ETS outside of the home are actually first-hand smoking variants which associate with ETS due to association of first-hand smoking with ETS.

The large negative MVMR estimates additionally illustrate why MVMR cannot be used to adjust for first hand smoking were this approach to have been included in the main analysis. If the variants associate with ETS via smoking, then there should not be any association of the variants with ETS conditional on smoking. As such, any resulting estimate would be liable to a large amount of conditional weak instrument bias.

The second table shows the effects for ETS exposure at home. No SNPs were associated with this phenotype at genome-wide significance, but one was at an indicative threshold (p < 5 x 10^-7^). Again, the effect estimate is technically consistent with the effects of rs16969968, but this is more due to the wide 95% CI. Since there was only one variant (rs62270410, an intron) associated with ETS at home we additionally ran a PHEWAS in OpenGWAS of which traits are associated (p < x 10^-5^) with this SNP. This finds that the SNP is associated with a number of inflammation related traits (primary biliary cholangitis, celiac disease, Ruminococcus E sp900314705 abundance in stool, multiple sclerosis, white blood cell count, multiple sclerosis). One possibility then is that the variant does not associate with actual ETS exposure but the perception of the amount of ETS a participant is exposed to at home. However, visual inspection of the regional Manhattan plot implies that it does not colocalize with the phenotypes identified in the PHEWAS. The additionally SNP looks like a bit of an outlier in terms of its association with ETS in the gene region (see the locus plot bellow).

Thus, it appears that the SNPs being identified with ETS exposure outside of the home are actually instruments for first hand smoking, while it is not clear why the SNP associated (at sub-genome-wide significance) with ETS exposure at home is associated with it. Given this, we would not be confident in the results of an analysis using these variants.


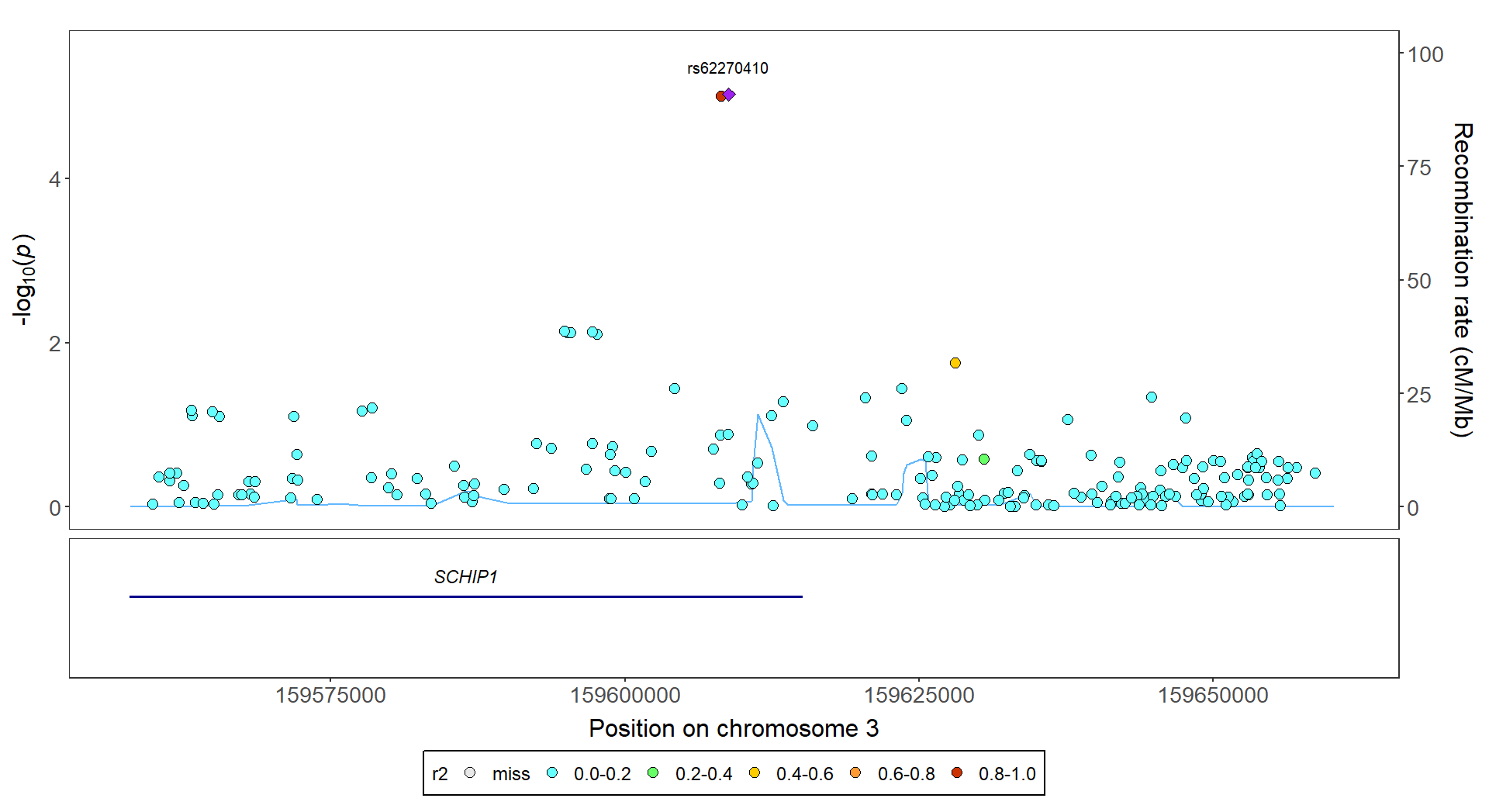


Extension to siblings

The approach outlined here could plausibly be extended to study the effects between sibling pairs. However, there are currently no GWAS of sibling smoking and it is unclear if sibling smoking can be robustly instrumented. In addition, since siblings both inherit smoking variants from their parents, an MVMR model including only each sibling’s genetic liability to smoke could be confounded by parental effects. As such, the model would have to include the parental liability to smoke. Similar to the comment with respect to trio modelling above, we would not have confidence with the parental smoking GWASs currently available to reliably estimate this.

References

1. Angrist JD, Pischke JS. Mostly Harmless Econometrics: An Empiricist’s Companion. Illustrated edition. Princeton: Princeton University Press; 2009. 392 p.

2. Burgess S, Davies NM, Thompson SG. Instrumental Variable Analysis with a Nonlinear Exposure–Outcome Relationship. Epidemiology. 2014 Nov;25(6):877–85.

3. Burgess S, Daniel RM, Butterworth AS, Thompson SG, the EPIC-InterAct Consortium. Network Mendelian randomization: using genetic variants as instrumental variables to investigate mediation in causal pathways. International Journal of Epidemiology. 2015 Apr 1;44(2):484–95.

4. Burgess S, Thompson SG. Multivariable Mendelian randomization: the use of pleiotropic genetic variants to estimate causal effects. Am J Epidemiol. 2015 Feb 15;181(4):251–60.

5. Collins R. What makes UK Biobank special. Lancet. 2012 Mar 1;379(9822):1173–4.

6. Howard DM, Adams MJ, Clarke TK, Hafferty JD, Gibson J, Shirali M, et al. Genome-wide meta-analysis of depression identifies 102 independent variants and highlights the importance of the prefrontal brain regions.: Supplementary Information [Internet]. Genetics; 2018 Oct [cited 2019 Apr 17]. Available from: http://biorxiv.org/lookup/doi/10.1101/433367

7. Wang Y, McKay JD, Rafnar T, Wang Z, Timofeeva MN, Broderick P, et al. Rare variants of large effect in BRCA2 and CHEK2 affect risk of lung cancer. Nat Genet. 2014 Jul;46(7):736–41.

8. Kimberley Burrows PH. data.bris. 2021 [cited 2022 Jun 28]. Genome-wide Association Study of Cancer Risk in UK Biobank. Available from: https://data.bris.ac.uk/data/dataset/aed0u12w0ede20olb0m77p4b9

9. Mishra A, Malik R, Hachiya T, Jürgenson T, Namba S, Posner DC, et al. Stroke genetics informs drug discovery and risk prediction across ancestries. Nature. 2022 Nov;611(7934):115–23.

10. Aragam KG, Jiang T, Goel A, Kanoni S, Wolford BN, Atri DS, et al. Discovery and systematic characterization of risk variants and genes for coronary artery disease in over a million participants. Nat Genet. 2022 Dec;54(12):1803–15.

11. FinnGen. FinnGen Documentation of R5 release. 2021; Available from: https://finngen.gitbook.io/documentation/v/r5/

12. Kurki MI, Karjalainen J, Palta P, Sipilä TP, Kristiansson K, Donner KM, et al. FinnGen provides genetic insights from a well-phenotyped isolated population. Nature. 2023 Jan;613(7944):508–18.

13. Boyd A, Golding J, Macleod J, Lawlor DA, Fraser A, Henderson J, et al. Cohort Profile: the ’children of the 90s’--the index offspring of the Avon Longitudinal Study of Parents and Children. Int J Epidemiol. 2013 Feb;42(1):111–27.

14. Fraser A, Macdonald-Wallis C, Tilling K, Boyd A, Golding J, Davey Smith G, et al. Cohort Profile: the Avon Longitudinal Study of Parents and Children: ALSPAC mothers cohort. Int J Epidemiol. 2013 Feb;42(1):97–110.

15. Woolf B, Sallis H, Munafo M, Gill D. Deriving GWAS summary estimates for paternal smoking in UK Biobank: A GWAS by subtraction [Internet]. OSF Preprints; 2023 [cited 2023 Mar 27]. Available from: https://osf.io/9yeu4/

16. Woolf B, Gill D, Munafò M, Burgess S. Reassessing the validity of using weighted linear models to implement multi-generational GWAS-by-subtraction: a response to Evans et al. BMC Res Notes. 2025 Jun 26;18:248.

17. Wootton RE, Richmond RC, Stuijfzand BG, Lawn RB, Sallis HM, Taylor GMJ, et al. Evidence for causal effects of lifetime smoking on risk for depression and schizophrenia: a Mendelian randomisation study. Psychol Med. 2020 Oct;50(14):2435–43.

18. Howard DM, Adams MJ, Shirali M, Clarke TK, Marioni RE, Davies G, et al. Genome-wide association study of depression phenotypes in UK Biobank identifies variants in excitatory synaptic pathways. Nat Commun. 2018 Apr 16;9:1470.

19. Wray NR, Ripke S, Mattheisen M, Trzaskowski M, Byrne EM, Abdellaoui A, et al. Genome-wide association analyses identify 44 risk variants and refine the genetic architecture of major depression. Nat Genet. 2018 May;50(5):668–81.

20. Kurki MI, Karjalainen J, Palta P, Sipilä TP, Kristiansson K, Donner K, et al. FinnGen: Unique genetic insights from combining isolated population and national health register data [Internet]. medRxiv; 2022 [cited 2022 Apr 14]. p. 2022.03.03.22271360. Available from: https://www.medrxiv.org/content/10.1101/2022.03.03.22271360v1

21. Ruth Mitchell E. data.bris. 2019 [cited 2022 Mar 12]. MRC IEU UK Biobank GWAS pipeline version 2. Available from: https://data.bris.ac.uk/data/dataset/pnoat8cxo0u52p6ynfaekeigi

22. Loh PR, Tucker G, Bulik-Sullivan BK, Vilhjálmsson BJ, Finucane HK, Salem RM, et al. Efficient Bayesian mixed-model analysis increases association power in large cohorts. Nat Genet. 2015 Mar;47(3):284–90.

23. Hyde CL, Nagle MW, Tian C, Chen X, Paciga SA, Wendland JR, et al. Identification of 15 genetic loci associated with risk of major depression in individuals of European descent. Nature genetics. 2016 Sep;48(9):1031.

24. Zhou W, Kanai M, Wu KHH, Rasheed H, Tsuo K, Hirbo JB, et al. Global Biobank Meta-analysis Initiative: Powering genetic discovery across human disease. Cell Genom. 2022 Oct 12;2(10):100192.

25. Risteys FinnGen R6 - I9_CVD [Internet]. [cited 2022 Apr 6]. Available from: https://r6.risteys.finngen.fi/phenocode/I9_CVD

26. Reed ZE, Wootton RE, Khouja JN, Richardson TG, Sanderson E, Davey Smith G, et al. Exploring pleiotropy in Mendelian randomisation analyses: What are genetic variants associated with ‘cigarette smoking initiation’ really capturing? Genetic Epidemiology. 2025;49(1):e22583.

27. Higgins J, Thomas J, Chandler J, Cumpston M, Li T, Page M, et al. Cochrane Handbook for Systematic Reviews of Interventions version 6.3 (updated February 2022) [Internet]. Cochrane; 2022. Available from: www.training.cochrane.org/handbook.

28. Hemani G, Zheng J, Elsworth B, Wade KH, Haberland V, Baird D, et al. The MR-Base platform supports systematic causal inference across the human phenome. eLife. 2018 May 30;7:e34408.

29. Slob EAW, Burgess S. A comparison of robust Mendelian randomization methods using summary data. Genetic Epidemiology. 2020;44(4):313–29.

30. Bowden J, Del Greco M F, Minelli C, Davey Smith G, Sheehan NA, Thompson JR. Assessing the suitability of summary data for two-sample Mendelian randomization analyses using MR-Egger regression: the role of the I2 statistic. Int J Epidemiol. 2016;45(6):1961–74.

31. Bowden J, Davey Smith G, Burgess S. Mendelian randomization with invalid instruments: effect estimation and bias detection through Egger regression. International Journal of Epidemiology. 2015 Apr 1;44(2):512–25.

32. He W, Xiong J, Yi GY. SIMEX R Package for Accelerated Failure Time Models with Covariate Measurement Error. Journal of Statistical Software. 2012 Jan 25;46:1–14.

33. Wu Y, Kang H, Ye T. Debiased Multivariable Mendelian Randomization [Internet]. arXiv; 2024 [cited 2024 Jul 23]. Available from: http://arxiv.org/abs/2402.00307

34. MendelianRandomization v0.9.0: updates to ... | Wellcome Open Research [Internet]. [cited 2024 Jul 23]. Available from: https://wellcomeopenresearch.org/articles/8-449#ref-20

35. Wang J, Zhao Q, Bowden J, Hemani G, Smith GD, Small DS, et al. Causal inference for heritable phenotypic risk factors using heterogeneous genetic instruments. PLOS Genetics. 2021 Jun 22;17(6):e1009575.

36. Sanderson E, Davey Smith G, Windmeijer F, Bowden J. An examination of multivariable Mendelian randomization in the single-sample and two-sample summary data settings. International Journal of Epidemiology. 2019 Jun 1;48(3):713–27.

37. Spiga F, Gibson M, Dawson S, Davey Smith G, Munafò MR, Higgins JP. Tools for the assessment of quality and risk of bias in Mendelian randomization studies: a systematic review [Internet]. medRxiv; 2021 [cited 2022 Jun 28]. p. 2021.10.21.21265126. Available from: https://www.medrxiv.org/content/10.1101/2021.10.21.21265126v1

38. Higgins JP, Savović J, Page MJ, Elbers RG, Sterne JA. Assessing risk of bias in a randomized trial. In: Cochrane Handbook for Systematic Reviews of Interventions [Internet]. John Wiley & Sons, Ltd; 2019 [cited 2022 May 16]. p. 205–28. Available from: https://onlinelibrary.wiley.com/doi/abs/10.1002/9781119536604.ch8

39. Howe LJ, Nivard MG, Morris TT, Hansen AF, Rasheed H, Cho Y, et al. Within-sibship GWAS improve estimates of direct genetic effects [Internet]. bioRxiv; 2021 [cited 2022 Mar 12]. p. 2021.03.05.433935. Available from: https://www.biorxiv.org/content/10.1101/2021.03.05.433935v1

40. Bulik-Sullivan BK, Loh PR, Finucane HK, Ripke S, Yang J, Patterson N, et al. LD Score regression distinguishes confounding from polygenicity in genome-wide association studies. Nat Genet. 2015 Mar;47(3):291–5.

41. Zhao Q, Chen Y, Wang J, Small DS. Powerful three-sample genome-wide design and robust statistical inference in summary-data Mendelian randomization. Int J Epidemiol. 2019 Oct 1;48(5):1478–92.

42. Sanderson E, Spiller W, Bowden J. Testing and correcting for weak and pleiotropic instruments in two-sample multivariable Mendelian randomization. Statistics in Medicine. 2021;40(25):5434–52.

43. Woolf B, Gill D, Grant AJ, Burgess S. MVMRmode: Introducing an R package for plurality valid estimators for multivariable Mendelian randomisation. PLoS One. 2024 May 7;19(5):e0291183.

44. Schwarzer G, Carpenter JR, Rücker G. Meta-Analysis with R [Internet]. Cham: Springer International Publishing; 2015 [cited 2022 Apr 14]. (Use R!). Available from: http://link.springer.com/10.1007/978-3-319-21416-0

45. Yavorska OO, Burgess S. MendelianRandomization: an R package for performing Mendelian randomization analyses using summarized data. Int J Epidemiol. 2017 01;46(6):1734–9.

46. R Core Team. R: A language and environment for statistical computing. R Foundation for Statistical Computin [Internet]. 2021. Available from: https://www.R-project.org/

47. Elsworth B, Lyon M, Alexander T, Liu Y, Matthews P, Hallett J, et al. The MRC IEU OpenGWAS data infrastructure [Internet]. bioRxiv; 2020 [cited 2022 Mar 30]. p. 2020.08.10.244293. Available from: https://www.biorxiv.org/content/10.1101/2020.08.10.244293v1

48. Woolf B, Cronjé HT, Zagkos L, Larsson SC, Gill D, Burgess S. Comparison of caffeine consumption behavior with plasma caffeine levels as exposure measures in drug-target Mendelian randomization. American Journal of Epidemiology. 2024 Jun 20;kwae143.

49. Woolf B, Yarmolinsky J, Gill D. Why exposure misidentification is a pervasive pitfall of Mendelian randomization studies with medication use as the exposure. International Journal of Epidemiology. 2025 Apr 1;54(2):dyaf031.

50. Sanderson E, Rosoff D, Palmer T, Tilling K, Smith GD, Hemani G. Heritable confounding in Mendelian randomization studies: structure, consequences and relevance for gene-environment equivalence [Internet]. medRxiv; 2025 [cited 2025 Nov 2]. p. 2024.09.05.24312293. Available from: https://www.medrxiv.org/content/10.1101/2024.09.05.24312293v3

51. Lassi G, Taylor AE, Timpson NJ, Kenny PJ, Mather RJ, Eisen T, et al. The CHRNA5–A3–B4 Gene Cluster and Smoking: From Discovery to Therapeutics. Trends Neurosci. 2016 Dec;39(12):851–61.
