# Supplementary material for: Assessing the causal effects of environmental tobacco smoke exposure: a meta-analytic Mendelian randomization study
Source: Nicotine Tob Res. 2026 Feb 25;28(8):1293–303. doi: 10.1093/ntr/ntag047 (PMC13389530; doi:10.1093/ntr/ntag047)
Supplement: Supplementary_Material_ntag047 [file supplementary_material_ntag047.zip › PS_Supplementary_Results_MM_bw_ntag047.docx]

**Supplementary Results**

Results of individual MR approaches

The results of the MR approaches using the IVW estimator are presented in Figure 3 , and provided in full in Supplementary Table S2. A risk of bias evaluation for each approach stratified by outcome can be found in Supplementary Table S3 and the Supplementary Results. Although the estimates across weak instrument robust estimators tended to be consistent, they had very wide 95% confidence intervals and conditional F statistics were all low. All studies were therefore placed at unclear risk of bias because of potential weak instrument bias.

Risk of bias in MR approaches

The risk of bias judgments, with supporting evidence for each approach and outcome, are presented in Supplementary Table S3 and summarised here:

*Relevance assumption.* All designs showed evidence of conditional weak instrument bias. For outcomes other than lung cancer and COPD, there was high variability in the estimates from the weak-instrument robust estimators. However, this is due likely due to imprecision as they were reasonably consistent in implying null effects. For lung cancer and COPD, the estimates were still variable but more consistent across estimators for the more precise the IVW estimates were. All MR approaches were therefore graded as unclear risk of bias.

*Independence assumption.* All GWAS scans used had implemented some type of control for population structure, and none of the instruments were associated with hair colour in the negative control (Supplementary Table S5). All MR approaches were therefore at low risk of bias.

*Exclusion restriction assumption.* Although Cochrane’s Q statistic implied there was evidence of pleiotropy for most MR approaches in most outcomes, the ‘pleiotropy robust’ estimators all provided consistent estimates. Based on this triangulation, MR approaches were with evidence of pleiotropy were kept at low risk of bias for this domain.

*Harmonisation.* Effect alleles were harmonised in all MR approaches so that effect estimates were in the same direction as the primary exposure GWAS. In addition, we allowed TwoSampleMR to remove palindromic SNPs which could not be aligned based on their minor allele frequency. All MR approaches were therefore at low risk of bias in this domain.

*Same population assumption.* All samples were drawn from demographically similar GWAS. Specifically, all the exposure GWAS scans were derived from either the UKB or ASLAPC. The fixation index for ASLAPC and UKB is less than 0.001 implying that they are from a homogeneous population. All outcome GWASs included data from the UKB and demographically similar (based on age, sex, and ethnicity) and can therefore be regarded as being sampled from similar populations.

*Clumping.* All genetic instruments were clumped at an r^2^ of 0.001 and KB of 10,000 and so all MR approaches were at low risk of bias in this domain.

*Winner’s Curse.* Although the same samples were used to select instruments and to estimate variant-exposure associations, we used the FDR Inverse Quantile Transformation Winner’s Curse correction on all exposure GWAS scans (32). All MR approaches were therefore classified as low risk of bias in this domain.

*Other risk of bias.* There was no clear evidence of a difference in MR estimates between the chip adjusted and no-chip adjusted analysis (Supplementary Table S4). All MR approaches were therefore kept at low risk of bias.
