# Supplementary material for: Assessing the causal effects of environmental tobacco smoke exposure: a meta-analytic Mendelian randomization study
Source: Nicotine Tob Res. 2026 Feb 25;28(8):1293–303. doi: 10.1093/ntr/ntag047 (PMC13389530; doi:10.1093/ntr/ntag047)
Supplement: Supplementary_Material_ntag047 [file supplementary_material_ntag047.zip › PS_Supplementary_Table_S1_MM_bw_ntag047.docx]

**Supplementary Table S1: Six pairwise comparisons of each of the MR approaches with rationale for the assumption of independence between the MR estimation approaches.**

| MR estimation approach | Maternal smoking on index individual’s outcome (Figure 2.A1) | Maternal smoking on paternal outcomes (Figure 2.A3) | Paternal smoking on maternal outcomes (Figure 2.A4) | Paternal smoking on index individual’s outcome (Figure 2.A2) |
| --- | --- | --- | --- | --- |
| Maternal smoking on index individual’s outcome (Figure 2.A1) | -- | No outcome sample overlap^2^ | No outcome sample overlap^2^ | Uncertain if independent^3^ |
| Maternal smoking on paternal outcomes (Figure 2.A3) | -- | -- | Factorial experiment and no outcome sample overlap^1^ | Factorial experiment and no outcome sample overlap^1^ |
| Paternal smoking on maternal outcomes  (Figure 2.A4) | -- | -- | -- | No outcome sample overlap^2^ |
| Paternal smoking on index individual’s outcome (Figure 2.A2) | -- | -- | -- | -- |

For a meta-analysis to be valid the estimates must be independent of each other. This requires that either the instruments are uncorrelated, the genetically predicted exposures are conditionally independent (by adjustment in the MVMR model), or the outcome samples are independent. The first and second are analogous to a factorial experiment in which people are independently randomised to two separate interventions with the same intended therapeutic effect (e.g., two different drugs that lower blood pressure). The third is equivalent to the assumption of no participant overlap made in traditional meta-analyses of randomised controlled trials (the standard error of a Wald ratio is the standard error from the outcome GWAS scaled by the effect in the exposure GWAS, so uncertainty in the exposure GWAS does not contribute to uncertainty in MR estimates given strong instruments). Here independence was only questionable for the pair with the two approaches assessing parental smoking and the index individual outcomes. This is because assortative mating and the social transmissibility of smoking mean that one parent’s genetically predicted smoking might predict the other parent’s liability to smoke.

^1^ The instruments used in each estimation approach were, by design, conditionally independent of each other (Figure 2), so this is analogous to a factorial experiment in which the same people are independently randomised to sperate interventions. In addition, the individuals in the phenotypes in the outcome GWASs were measured in independent people.

^2^ The outcome individuals being measured cannot overlap, for example mothers and fathers (because someone cannot be both a mother and a father to the same individual). In addition, because the outcome individual’s smoking has been adjusted for, there should not be a correlation due to assortative mating.

^3^ These analyses have the same people. Maternal and paternal instruments should be independent of each other if we assume no assortative mating. Because we are conditioning on the offspring’s genotype, this should also adjust for some genetic overlap due to assortative mating among inherited variants. However, because children only inherit a random 50% of each parent’s genetic variation, there could still be some residual correlation due to assortative mating after adjusting for the offspring’s genotype.
