# Supplementary material for: Assessing the causal effects of environmental tobacco smoke exposure: a meta-analytic Mendelian randomization study
Source: Nicotine Tob Res. 2026 Feb 25;28(8):1293–303. doi: 10.1093/ntr/ntag047 (PMC13389530; doi:10.1093/ntr/ntag047)
Supplement: Supplementary_Material_ntag047 [file supplementary_material_ntag047.zip › PS_Supplementary_Table_S2_ntag047.docx]

Supplementary Table S2.a: Full results of MR analyses when lung cancer is the outcome

| **Description** | **IVW Beta** | **IVW**  **SE** | **IVW p** | **Egger Beta** | **Egger SE** | **Egger**  **p** | **Median Beta** | **Median SE** | **Median**  **p** | **Mode Beta** | **Mode**  **LCI** | **Mode**  **UCI** | **Mode**  **p** | **N SNP** | **F exposure 1** | **F exposure 2** | **Egger**  **Intercept** | **Egger**  **Intercept**  **SE** | **Egger**  **Intercept**  **p** | **Q**  **Statistic** | **Q p** | **Debias**  **IVW**  **beta** | **Debias**  **IVW**  **se** | **Grapple**  **beta** | **Grapple**  **se** | **Qhet**  **beta** | **mvGMM**  **beta** | **mvGMM**  **se** | **Qhet**  **CI** |
| --- | --- | --- | --- | --- | --- | --- | --- | --- | --- | --- | --- | --- | --- | --- | --- | --- | --- | --- | --- | --- | --- | --- | --- | --- | --- | --- | --- | --- | --- |
| mum smoking -> index | 0.626 | 1.110 | 0.574 | 1.367 | 1.122 | 0.226 | 2.274 | 0.818 | 0.005 | 1.709 | 0.350 | 3.023 | 0.011 | 120 | 1.7 | 1.9 | -0.019 | 0.007 | 0.012 | 406.385 | < 0.001 | 0.263 | 1.501 | 2.874 | 1.736 | 0.860 | 0.865 | 1.122 | -2.494-2.753 |
| dad smoking -> index | 0.154 | 0.241 | 0.523 | 0.010 | 0.336 | 0.977 | 0.196 | 0.089 | 0.027 | 0.045 | -0.260 | 0.386 | 0.708 | 116 | 0.1 | 6.0 | 0.002 | 0.003 | 0.537 | 372.767 | < 0.001 | -0.022 | 0.019 | 52.072 | 5606.429 | 0.073 | 1.058 | 0.298 | -0.458-0.621 |
| mum smoking -> dad | 2.299 | 0.548 | 0.001 | 5.512 | 1.900 | 0.010 | 2.049 | 0.464 | < 0.001 | 2.844 | 1.097 | 3.568 | 0.003 | 19 | 6.1 | 0.1 | -0.029 | 0.017 | 0.098 | 6.022 | 0.988 | 2.350 | 0.271 | 2.530 | 4.941 | 2.416 | 2.416 | 1.007 | 1.411-3.382 |
| dad smoking -> mum | 1.104 | 0.673 | 0.119 | 1.856 | 1.027 | 0.090 | 0.279 | 0.259 | 0.281 | 0.754 | -1.080 | 1.851 | 0.024 | 19 | 0.1 | 6.1 | -0.008 | 0.008 | 0.346 | 7.472 | 0.963 | -0.083 | 0.037 | 8.760 | 101.620 | 1.235 | 1.235 | 1.122 | 0.212-2.713 |
| index smoke -> index | 1.607 | 0.159 | < 0.001 | 3.993 | 0.637 | < 0.001 | 1.160 | 0.132 | < 0.001 | 0.582 | -0.185 | 1.349 | 0.140 | 113 | 42.7 | NA | -0.036 | 0.009 | 0.000 | 418.496 | < 0.001 | NA | NA | NA | NA | NA | NA | NA | NA |

Supplementary Table S2.b: Full results of MR analyses when chronic obstructive pulmonary diseases is the outcome

| **Description** | **IVW Beta** | **IVW**  **SE** | **IVW p** | **Egger Beta** | **Egger SE** | **Egger**  **p** | **Median Beta** | **Median SE** | **Median**  **p** | **Mode Beta** | **Mode**  **LCI** | **Mode**  **UCI** | **Mode**  **p** | **N SNP** | **F exposure 1** | **F exposure 2** | **Egger**  **Intercept** | **Egger**  **Intercept**  **SE** | **Egger**  **Intercept**  **p** | **Q**  **Statistic** | **Q p** | **Debias**  **IVW**  **beta** | **Debias**  **IVW**  **se** | **Grapple**  **beta** | **Grapple**  **se** | **Qhet**  **beta** | **mvGMM**  **beta** | **mvGMM**  **se** | **Qhet**  **CI** |
| --- | --- | --- | --- | --- | --- | --- | --- | --- | --- | --- | --- | --- | --- | --- | --- | --- | --- | --- | --- | --- | --- | --- | --- | --- | --- | --- | --- | --- | --- |
| mum smoking -> index | 0.479 | 0.725 | 0.510 | 0.821 | 0.748 | 0.275 | 0.672 | 0.586 | 0.251 | 0.791 | -0.063 | 1.688 | 0.067 | 100 | 1.5 | 1.8 | -0.006 | 0.004 | 0.106 | 269.721 | < 0.001 | 0.007 | 1.299 | 1.061 | 1.373 | 0.850 | 0.633 | 0.691 | -0.485-2.122 |
| dad smoking -> index | 0.125 | 0.137 | 0.364 | 0.071 | 0.175 | 0.685 | 0.089 | 0.060 | 0.138 | 0.079 | -0.112 | 0.274 | 0.196 | 100 | 0.2 | 6.2 | 0.001 | 0.002 | 0.623 | 232.328 | < 0.001 | -0.021 | 0.014 | 16.812 | 758.362 | 0.077 | -2.749 | 0.722 | -0.347-0.402 |
| mum smoking -> dad | 1.722 | 0.369 | 0.000 | 3.139 | 1.350 | 0.034 | 1.569 | 0.387 | < 0.001 | 1.104 | 0.613 | 2.198 | 0.000 | 19 | 6.1 | 0.1 | -0.013 | 0.012 | 0.291 | 6.569 | 0.981 | 1.763 | 0.238 | 1.787 | 3.785 | 1.872 | 0.978 | 0.822 | 1.175-2.75 |
| dad smoking -> mum | 0.637 | 0.349 | 0.085 | 0.112 | 0.519 | 0.833 | 0.342 | 0.166 | 0.039 | 0.637 | -0.018 | 1.292 | 0.042 | 19 | 0.1 | 6.1 | 0.005 | 0.004 | 0.199 | 4.986 | 0.996 | -0.048 | 0.029 | 3.621 | 26.134 | 0.921 | 0.921 | 0.861 | 0.313-1.657 |
| index smoke -> index | 1.426 | 0.091 | < 0.001 | 2.336 | 0.344 | < 0.001 | 1.256 | 0.093 | < 0.001 | 1.342 | 0.899 | 1.785 | < 0.001 | 95 | 43.3 | NA | -0.014 | 0.005 | 0.008 | 277.655 | < 0.001 | NA | NA | NA | NA | NA | NA | NA | NA |

Supplementary Table S2.c: Full results of MR analyses when hypertension is the outcome

| **Description** | **IVW Beta** | **IVW**  **SE** | **IVW p** | **Egger Beta** | **Egger SE** | **Egger**  **p** | **Median Beta** | **Median SE** | **Median**  **p** | **Mode Beta** | **Mode**  **LCI** | **Mode**  **UCI** | **Mode**  **p** | **N SNP** | **F exposure 1** | **F exposure 2** | **Egger**  **Intercept** | **Egger**  **Intercept**  **SE** | **Egger**  **Intercept**  **p** | **Q**  **Statistic** | **Q p** | **Debias**  **IVW**  **beta** | **Debias**  **IVW**  **se** | **Grapple**  **beta** | **Grapple**  **se** | **Qhet**  **beta** | **mvGMM**  **beta** | **mvGMM**  **se** | **Qhet**  **CI** |
| --- | --- | --- | --- | --- | --- | --- | --- | --- | --- | --- | --- | --- | --- | --- | --- | --- | --- | --- | --- | --- | --- | --- | --- | --- | --- | --- | --- | --- | --- |
| mum smoking -> index | -0.231 | 0.433 | 0.595 | -0.446 | 0.448 | 0.322 | 0.125 | 0.336 | 0.710 | 0.743 | 0.258 | 1.391 | < 0.001 | 128 | 1.6 | 1.9 | 0.004 | 0.003 | 0.088 | 696.393 | < 0.001 | -0.954 | 0.544 | 0.031 | 1.262 | -0.174 | -0.202 | 0.444 | -1.41-0.707 |
| dad smoking -> index | -0.117 | 0.078 | 0.138 | -0.095 | 0.107 | 0.376 | 0.042 | 0.036 | 0.244 | -0.019 | -0.132 | 0.112 | 0.434 | 127 | 0.2 | 7.8 | 0.000 | 0.001 | 0.764 | 443.489 | < 0.001 | 0.024 | 0.007 | -7.830 | 129.805 | -0.104 | -0.812 | 0.178 | -0.272-0.098 |
| mum smoking -> dad | -0.137 | 0.216 | 0.534 | -0.539 | 0.811 | 0.515 | -0.033 | 0.255 | 0.896 | -0.330 | -1.259 | 1.051 | 0.368 | 19 | 6.1 | 0.1 | 0.004 | 0.007 | 0.613 | 13.721 | 0.620 | -0.146 | 0.145 | -0.223 | 3.003 | -0.188 | -0.188 | 0.261 | -0.71-0.353" |
| dad smoking -> mum | -0.103 | 0.253 | 0.690 | 0.608 | 0.321 | 0.076 | -0.067 | 0.097 | 0.489 | -0.037 | -0.390 | 0.301 | 0.817 | 19 | 0.1 | 6.1 | -0.007 | 0.002 | 0.010 | 31.207 | 0.013 | 0.008 | 0.013 | -8.088 | 591.473 | -0.040 | -0.880 | 0.768 | -0.586-0.301 |
| index smoke -> index | 0.263 | 0.062 | < 0.001 | -0.105 | 0.255 | 0.681 | 0.175 | 0.047 | 0.000 | 0.121 | -0.059 | 0.300 | 0.191 | 120 | 42.1 | NA | 0.006 | 0.004 | 0.139 | 720.186 | < 0.001 | NA | NA | NA | NA | NA | NA | NA | NA |

Supplementary Table S2.d: Full results of MR analyses when depression is the outcome

| **Description** | **IVW Beta** | **IVW**  **SE** | **IVW p** | **Egger Beta** | **Egger SE** | **Egger**  **p** | **Median Beta** | **Median SE** | **Median**  **p** | **Mode Beta** | **Mode**  **LCI** | **Mode**  **UCI** | **Mode**  **p** | **N SNP** | **F exposure 1** | **F exposure 2** | **Egger**  **Intercept** | **Egger**  **Intercept**  **SE** | **Egger**  **Intercept**  **p** | **Q**  **Statistic** | **Q p** | **Debias**  **IVW**  **beta** | **Debias**  **IVW**  **se** | **Grapple**  **beta** | **Grapple**  **se** | **Qhet**  **beta** | **mvGMM**  **beta** | **mvGMM**  **se** | **Qhet**  **CI** |
| --- | --- | --- | --- | --- | --- | --- | --- | --- | --- | --- | --- | --- | --- | --- | --- | --- | --- | --- | --- | --- | --- | --- | --- | --- | --- | --- | --- | --- | --- |
| mum smoking -> index | -0.017 | 0.415 | 0.967 | -0.134 | 0.438 | 0.761 | -0.102 | 0.325 | 0.753 | 0.203 | -0.325 | 0.693 | 0.291 | 124 | 1.6 | 1.9 | 0.002 | 0.002 | 0.406 | 539.676 | < 0.001 | -0.578 | 0.549 | -0.191 | 1.084 | 0.119 | 0.023 | 0.405 | -0.676-0.818 |
| dad smoking -> index | -0.039 | 0.075 | 0.601 | -0.066 | 0.099 | 0.506 | -0.018 | 0.034 | 0.599 | -0.065 | -0.149 | 0.027 | 0.051 | 122 | 0.2 | 6.0 | 0.000 | 0.001 | 0.678 | 499.185 | < 0.001 | 0.008 | 0.007 | -18.741 | 2117.651 | -0.072 | 0.638 | 0.144 | -0.263-0.018 |
| mum smoking -> dad | 0.220 | 0.418 | 0.606 | -1.448 | 1.524 | 0.356 | 0.319 | 0.516 | 0.537 | 0.301 | -0.583 | 1.145 | 0.512 | 19 | 6.1 | 0.1 | 0.015 | 0.013 | 0.272 | 19.513 | 0.243 | 0.231 | 0.370 | -0.233 | 128.650 | 0.245 | 0.245 | 0.570 | -0.692-1.026 |
| dad smoking -> mum | 0.678 | 0.429 | 0.132 | 0.868 | 0.670 | 0.214 | 0.677 | 0.198 | 0.001 | 0.678 | -0.224 | 1.494 | 0.002 | 19 | 0.1 | 6.1 | -0.002 | 0.005 | 0.713 | 6.718 | 0.978 | -0.051 | 0.026 | 4.738 | 44.525 | 0.257 | 1.080 | 0.965 | -0.621-1.377 |
| index smoke -> index | 0.491 | 0.058 | < 0.001 | 0.305 | 0.245 | 0.216 | 0.401 | 0.049 | < 0.001 | 0.303 | 0.077 | 0.530 | 0.010 | 116 | 41.9 | NA | 0.003 | 0.004 | 0.436 | 551.500 | < 0.001 | NA | NA | NA | NA | NA | NA | NA | NA |

Supplementary Table S2.e: Full results of MR analyses when coronary heart disease is the outcome

| **Description** | **IVW Beta** | **IVW**  **SE** | **IVW p** | **Egger Beta** | **Egger SE** | **Egger**  **p** | **Median Beta** | **Median SE** | **Median**  **p** | **Mode Beta** | **Mode**  **LCI** | **Mode**  **UCI** | **Mode**  **p** | **N SNP** | **F exposure 1** | **F exposure 2** | **Egger**  **Intercept** | **Egger**  **Intercept**  **SE** | **Egger**  **Intercept**  **p** | **Q**  **Statistic** | **Q p** | **Debias**  **IVW**  **beta** | **Debias**  **IVW**  **se** | **Grapple**  **beta** | **Grapple**  **se** | **Qhet**  **beta** | **mvGMM**  **beta** | **mvGMM**  **se** | **Qhet**  **CI** |
| --- | --- | --- | --- | --- | --- | --- | --- | --- | --- | --- | --- | --- | --- | --- | --- | --- | --- | --- | --- | --- | --- | --- | --- | --- | --- | --- | --- | --- | --- |
| mum smoking -> index | 0.174 | 0.397 | 0.662 | 0.071 | 0.414 | 0.864 | 0.421 | 0.352 | 0.232 | 0.542 | -0.300 | 1.222 | 0.079 | 128 | 1.6 | 1.9 | 0.002 | 0.002 | 0.374 | 427.706 | < 0.001 | 0.104 | 0.561 | 0.759 | 0.885 | 0.461 | 0.001 | 0.388 | -0.503-1.165 |
| dad smoking -> index | 0.092 | 0.072 | 0.205 | 0.168 | 0.098 | 0.090 | 0.090 | 0.035 | 0.010 | 0.118 | 0.022 | 0.209 | 0.001 | 127 | 0.2 | 7.8 | -0.001 | 0.001 | 0.256 | 334.347 | < 0.001 | -0.019 | 0.008 | 7.337 | 133.814 | 0.100 | -2.093 | 0.451 | -0.055-0.199 |
| mum smoking -> dad | -0.047 | 0.182 | 0.798 | 0.171 | 0.687 | 0.807 | 0.277 | 0.236 | 0.241 | 0.332 | -0.070 | 0.858 | 0.111 | 19 | 6.1 | 0.1 | -0.002 | 0.006 | 0.746 | 10.612 | 0.833 | -0.046 | 0.151 | -0.100 | 2.390 | -0.035 | -0.367 | 0.460 | -0.428-0.339 |
| dad smoking -> mum | -0.295 | 0.253 | 0.260 | -0.045 | 0.388 | 0.910 | -0.416 | 0.106 | < 0.001 | -0.299 | -0.676 | 0.079 | 0.114 | 19 | 0.1 | 6.1 | -0.003 | 0.003 | 0.406 | 11.220 | 0.796 | 0.022 | 0.016 | -4.165 | 78.921 | -0.100 | -1.136 | 0.987 | -0.676-0.391 |
| index smoke -> index | 0.459 | 0.054 | < 0.001 | 0.147 | 0.223 | 0.513 | 0.481 | 0.052 | < 0.001 | 0.574 | 0.309 | 0.839 | < 0.001 | 120 | 42.1 | NA | 0.005 | 0.003 | 0.152 | 398.778 | < 0.001 | NA | NA | NA | NA | NA | NA | NA | NA |

Supplementary Table S2.f: Full results of MR analyses when stroke is the outcome

| **Description** | **IVW Beta** | **IVW**  **SE** | **IVW p** | **Egger Beta** | **Egger SE** | **Egger**  **p** | **Median Beta** | **Median SE** | **Median**  **p** | **Mode Beta** | **Mode**  **LCI** | **Mode**  **UCI** | **Mode**  **p** | **N SNP** | **F exposure 1** | **F exposure 2** | **Egger**  **Intercept** | **Egger**  **Intercept**  **SE** | **Egger**  **Intercept**  **p** | **Q**  **Statistic** | **Q p** | **Debias**  **IVW**  **beta** | **Debias**  **IVW**  **se** | **Grapple**  **beta** | **Grapple**  **se** | **Qhet**  **beta** | **mvGMM**  **beta** | **mvGMM**  **se** | **Qhet**  **CI** |
| --- | --- | --- | --- | --- | --- | --- | --- | --- | --- | --- | --- | --- | --- | --- | --- | --- | --- | --- | --- | --- | --- | --- | --- | --- | --- | --- | --- | --- | --- |
| mum smoking -> index | -0.194 | 0.381 | 0.611 | -0.101 | 0.404 | 0.803 | 0.140 | 0.470 | 0.766 | -0.297 | -0.955 | 0.360 | 0.376 | 129 | 1.7 | 1.9 | -0.002 | 0.002 | 0.480 | 162.320 | 0.014 | 6.047 | 97.711 | -2.204 | 2.650 | -0.241 | -0.242 | 0.383 | -1.026-0.52 |
| dad smoking -> index | 0.140 | 0.067 | 0.039 | 0.182 | 0.091 | 0.048 | 0.231 | 0.046 | < 0.001 | 0.140 | 0.011 | 0.269 | 0.008 | 127 | 0.2 | 7.8 | -0.001 | 0.001 | 0.499 | 128.485 | 0.373 | -0.030 | 0.013 | 3.921 | 23.718 | 0.211 | 0.211 | 0.075 | 0.02-0.366 |
| mum smoking -> dad | -0.078 | 0.231 | 0.741 | -1.052 | 0.836 | 0.227 | -0.092 | 0.304 | 0.763 | -0.372 | -1.102 | 0.780 | 0.654 | 19 | 6.1 | 0.1 | 0.009 | 0.007 | 0.244 | 6.376 | 0.984 | -0.074 | 0.197 | -0.156 | 2.265 | -0.063 | -0.412 | 0.911 | -0.522-0.366 |
| dad smoking -> mum | -0.276 | 0.255 | 0.295 | 0.120 | 0.379 | 0.755 | -0.402 | 0.120 | 0.001 | -0.276 | -0.754 | 0.202 | 0.160 | 19 | 0.1 | 6.1 | -0.004 | 0.003 | 0.185 | 10.976 | 0.811 | < 0.001 | < 0.001 | -4.069 | 73.055 | 0.036 | -3.332 | 2.848 | -0.596-0.612 |
| index smoke -> index | 0.271 | 0.052 | < 0.001 | 0.290 | 0.216 | 0.182 | 0.287 | 0.071 | < 0.001 | 0.177 | -0.158 | 0.512 | 0.303 | 120 | 42.1 | NA | 0.000 | 0.003 | 0.929 | 149.078 | 0.032 | NA | NA | NA | NA | NA | NA | NA | NA |
