# Supplementary material for: Assessing the causal effects of environmental tobacco smoke exposure: a meta-analytic Mendelian randomization study
Source: Nicotine Tob Res. 2026 Feb 25;28(8):1293–303. doi: 10.1093/ntr/ntag047 (PMC13389530; doi:10.1093/ntr/ntag047)
Supplement: Supplementary_Material_ntag047 [file supplementary_material_ntag047.zip › PS_Supplementary_Table_S3_ROB_MM_bw_ntag047.docx]

**Supplementary Table S3a: Risk of bias for 4 MR estimation approaches when lung cancer is the outcome.**

| **MR estimation approach** | **IV1 – relevance** | **IV2 – independence** | | | | **IV3 – exclusion restriction** | **Harmonisation of SNP effects** | **Exposure-outcome GWAS from the same population^1^** | **Clumping of genetic instruments** | **Other risk of bias^2^** | **Winner’s Curse** | **Overall risk** |
| --- | --- | --- | --- | --- | --- | --- | --- | --- | --- | --- | --- | --- |
|  |  | **Hair colour negative control** | **GWAS covariates or LSDC intercept** | **Unadjusted pathways in DAG** | **Judgment** |  |  |  |  |  |  |  |
| Maternal smoking on index individual’s outcome | Unclear (conditional F stats are small. Although ‘robust’ estimators are consistent, their SEs are very wide) | Low (No evidence of association) | Low (All GWASs adjust for PCs or use BOLT-LMM or GC) | Low (adjust for index smoking) | Low | Low (Cochrane Q p-value < 0.001, but ‘robust’ estimators provide consistent estimates) | Low (data harmonised and palindromes removed when direction cannot be inferred from MAF) | Low (exposure and outcome GWASs both include UKB) | Low (r^2^ of 0.001 and KB of 10,000) | Low (non-differential measurement error^3^) | Low (use FIQT) | Unclear (unclear in at least one domain) |
| Paternal smoking on index individual’s outcome | Unclear (conditional F stats are small. Although ‘robust’ estimators are consistent, their SEs are very wide) | Low (No evidence of association) | Low (All GWASs adjust for PCs or use BOLT-LMM or GC) | Low (adjust for index smoking) | Low | Low (Cochrane Q p-value < 0.001, but ‘robust’ estimators provide consistent estimates) | Low (data harmonised and palindromes removed when direction cannot be inferred from MAF) | Low (ALSPAC and UKB sampled form homogeneous populations. Outcome includes UKB) | Low (r^2^ of 0.001 and KB of 10,000) | Low (none) | Low (use FIQT) | Unclear (unclear in at least one domain) |
| Maternal smoking on paternal outcomes | Unclear (conditional F stats are small. Although ‘robust’ estimators are consistent, their SEs are very wide) | Low (No evidence of association) | Low (All GWASs adjust for PCs or use BOLT-LMM or GC) | Low (adjust for dad smoking) | Low | Low (Cochrane Q p-value = 0.257) | Low (data harmonised and palindromes removed when direction cannot be inferred from MAF) | Low (ALSPAC and UKB sampled form homogeneous populations. Outcome includes UKB) | Low (r^2^ of 0.001 and KB of 10,000) | Low (non-differential measurement error^3^) | Low (use FIQT) | Unclear (unclear in at least one domain) |
| Paternal smoking on maternal outcomes | Unclear (conditional F stats are small. Although ‘robust’ estimators are consistent, their SEs are very wide) | Low (No evidence of association) | Low (All GWASs adjust for PCs or use BOLT-LMM or GC) | Low (adjust for mum smoking) | Low | Low (Cochrane Q p-value = 0.011, but ‘robust’ estimators provide consistent estimates) | Low (data harmonised and palindromes removed when direction cannot be inferred from MAF) | Low (ALSPAC and UKB sampled form homogeneous populations. Outcome includes UKB) | Low (r^2^ of 0.001 and KB of 10,000) | Low (non-differential measurement error^3^) | Low (use FIQT) | Unclear (unclear in at least one domain) |

**Supplementary Table S3b: Risk of bias for 4 MR estimation approaches when Chronic obstructive pulmonary disease is the outcome.**

| **MR estimation approach** | **IV1 – relevance** | **IV2 – independence** | | | | **IV3 – exclusion restriction** | **Harmonisation of SNP effects** | **Exposure-outcome GWAS from the same population^1^** | **Clumping of genetic instruments** | **Other risk of bias^2^** | **Winner’s Curse** | **Overall risk** |
| --- | --- | --- | --- | --- | --- | --- | --- | --- | --- | --- | --- | --- |
|  |  | **Hair colour negative control** | **GWAS covariates or LSDC intercept** | **Unadjusted pathways in DAG** | **Judgment** |  |  |  |  |  |  |  |
| Maternal smoking on index individual’s outcome | Unclear (conditional F stats are small. Although ‘robust’ estimators are consistent, their SEs are very wide) | Low (No evidence of association) | Low (All GWASs adjust for PCs or use BOLT-LMM or GC) | Low (adjust for index smoking) | Low | Low (Cochrane Q p-value < 0.001, but ‘robust’ estimators provide consistent estimates) | Low (data harmonised and palindromes removed when direction cannot be inferred from MAF) | Low (exposure and outcome GWASs both include UKB) | Low (r^2^ of 0.001 and KB of 10,000) | Low (non-differential measurement error^3^) | Low (use FIQT) | Unclear (unclear in at least one domain) |
| Paternal smoking on index individual’s outcome | Unclear (conditional F stats are small. Although ‘robust’ estimators are consistent, their SEs are very wide) | Low (No evidence of association) | Low (All GWASs adjust for PCs or use BOLT-LMM or GC) | Low (adjust for index smoking) | Low | Low (Cochrane Q p-value < 0.001, but ‘robust’ estimators provide consistent estimates) | Low (data harmonised and palindromes removed when direction cannot be inferred from MAF) | Low (ALSPAC and UKB sampled form homogeneous populations. Outcome includes UKB) | Low (r^2^ of 0.001 and KB of 10,000) | Low (none) | Low (use FIQT) | Unclear (unclear in at least one domain) |
| Maternal smoking on paternal outcomes | Unclear (conditional F stats are small. Although ‘robust’ estimators are consistent, their SEs are very wide) | Low (No evidence of association) | Low (All GWASs adjust for PCs or use BOLT-LMM or GC) | Low (adjust for dad smoking) | Low | Low (Cochrane Q p = 0.462) | Low (data harmonised and palindromes removed when direction cannot be inferred from MAF) | Low (ALSPAC and UKB sampled form homogeneous populations. Outcome includes UKB) | Low (r^2^ of 0.001 and KB of 10,000) | Low (non-differential measurement error^3^) | Low (use FIQT) | Unclear (unclear in at least one domain) |
| Paternal smoking on maternal outcomes | Unclear (conditional F stats are small. Although ‘robust’ estimators are consistent, their SEs are very wide) | Low (No evidence of association) | Low (All GWASs adjust for PCs or use BOLT-LMM or GC) | Low (adjust for mum smoking) | Low | Low (Cochrane Q p = 0.994) | Low (data harmonised and palindromes removed when direction cannot be inferred from MAF) | Low (ALSPAC and UKB sampled form homogeneous populations. Outcome includes UKB) | Low (r^2^ of 0.001 and KB of 10,000) | Low (non-differential measurement error^3^) | Low (use FIQT) | Unclear (unclear in at least one domain) |

**Supplementary Table S3c: Risk of bias for 4 MR estimation approaches when stroke is the outcome.**

| **MR estimation approach** | **IV1 – relevance** | **IV2 – independence** | | | | **IV3 – exclusion restriction** | **Harmonisation of SNP effects** | **Exposure-outcome GWAS from the same population^1^** | **Clumping of genetic instruments** | **Other risk of bias^2^** | **Winner’s curse** | **Overall risk** |
| --- | --- | --- | --- | --- | --- | --- | --- | --- | --- | --- | --- | --- |
|  |  | **Hair colour negative control** | **GWAS covariates or LSDC intercept** | **Unadjusted pathways in DAG** | **Judgment** |  |  |  |  |  |  |  |
| Maternal smoking on index individual’s outcome | Unclear (conditional F stats are small. Although ‘robust’ estimators are consistent, their SEs are very wide) | Low (No evidence of association) | Low (All GWASs adjust for PCs or use BOLT-LMM or GC) | Low (adjust for index smoking) | Low | Low (Cochrane Q p-value = 0.021, but ‘robust’ estimators provide consistent estimates) | Low (data harmonised and palindromes removed when direction cannot be inferred from MAF) | Low (exposure and outcome GWASs both include UKB) | Low (r^2^ of 0.001 and KB of 10,000) | Low (non-differential measurement error^3^) | Low (use FIQT) | Unclear (unclear in at least one domain) |
| Paternal smoking on index individual’s outcome | Unclear (conditional F stats are small. Although ‘robust’ estimators are consistent, their SEs are very wide) | Low (No evidence of association) | Low (All GWASs adjust for PCs or use BOLT-LMM or GC) | Low (adjust for index smoking) | Low | Low (Cochrane Q p-value = 0.023, but ‘robust’ estimators provide consistent estimates) | Low (data harmonised and palindromes removed when direction cannot be inferred from MAF) | Low (ALSPAC and UKB sampled form homogeneous populations. Outcome includes UKB) | Low (r^2^ of 0.001 and KB of 10,000) | Low (none) | Low (use FIQT) | Unclear (unclear in at least one domain) |
| Maternal smoking on paternal outcomes | Unclear (conditional F stats are small. Although ‘robust’ estimators are consistent, their SEs are very wide) | Low (No evidence of association) | Low (All GWASs adjust for PCs or use BOLT-LMM or GC) | Low (adjust for dad smoking) | Low | Low (Cochrane Q p = 0.339) | Low (data harmonised and palindromes removed when direction cannot be inferred from MAF) | Low (ALSPAC and UKB sampled form homogeneous populations. Outcome includes UKB) | Low (r^2^ of 0.001 and KB of 10,000) | Low (non-differential measurement error^3^) | Low (use FIQT) | Unclear (unclear in at least one domain) |
| Paternal smoking on maternal outcomes | Unclear (conditional F stats are small. Although ‘robust’ estimators are consistent, their SEs are very wide) | Low (No evidence of association) | Low (All GWASs adjust for PCs or use BOLT-LMM or GC) | Low (adjust for mum smoking) | Low | Low (Cochrane Q p = 0.077) | Low (data harmonised and palindromes removed when direction cannot be inferred from MAF) | Low (ALSPAC and UKB sampled form homogeneous populations. Outcome includes UKB) | Low (r^2^ of 0.001 and KB of 10,000) | Low (non-differential measurement error^3^) | Low (use FIQT) | Unclear (unclear in at least one domain) |

**Supplementary Table S3d: Risk of bias for 4 MR estimation approaches when coronary heart disease is the outcome.**

| **MR estimation approach** | **IV1 – relevance** | **IV2 – independence** | | | | **IV3 – exclusion restriction** | **Harmonisation of SNP effects** | **Exposure-outcome GWAS from the same population^1^** | **Clumping of genetic instruments** | **Other risk of bias^2^** | **Winner’s Curse** | **Overall risk** |
| --- | --- | --- | --- | --- | --- | --- | --- | --- | --- | --- | --- | --- |
|  |  | **Hair colour negative control** | **GWAS covariates or LSDC intercept** | **Unadjusted pathways in DAG** | **Judgment** |  |  |  |  |  |  |  |
| Maternal smoking on index individual’s outcome | Unclear (conditional F stats are small. Although ‘robust’ estimators are consistent, their SEs are very wide) | Low (No evidence of association) | Low (All GWASs adjust for PCs or use BOLT-LMM or GC) | Low (adjust for index smoking) | Low | Low (Cochrane Q p-value < 0.001, but ‘robust’ estimators provide consistent estimates) | Low (data harmonised and palindromes removed when direction cannot be inferred from MAF) | Low (exposure and outcome GWASs both include UKB) | Low (r^2^ of 0.001 and KB of 10,000) | Low (non-differential measurement error^3^) | Low (use FIQT) | Unclear (unclear in at least one domain) |
| Paternal smoking on index individual’s outcome | Unclear (conditional F stats are small. Although ‘robust’ estimators are consistent, their SEs are very wide) | Low (No evidence of association) | Low (All GWASs adjust for PCs or use BOLT-LMM or GC) | Low (adjust for index smoking) | Low | Low (Cochrane Q p-value < 0.001, but ‘robust’ estimators provide consistent estimates) | Low (data harmonised and palindromes removed when direction cannot be inferred from MAF) | Low (ALSPAC and UKB sampled form homogeneous populations. Outcome includes UKB) | Low (r^2^ of 0.001 and KB of 10,000) | Low (none) | Low (use FIQT) | Unclear (unclear in at least one domain) |
| Maternal smoking on paternal outcomes | Unclear (conditional F stats are small. Although ‘robust’ estimators are consistent, their SEs are very wide) | Low (No evidence of association) | Low (All GWASs adjust for PCs or use BOLT-LMM or GC) | Low (adjust for dad smoking) | Low | Low (Cochrane Q p = 0.443) | Low (data harmonised and palindromes removed when direction cannot be inferred from MAF) | Low (ALSPAC and UKB sampled form homogeneous populations. Outcome includes UKB) | Low (r^2^ of 0.001 and KB of 10,000) | Low (non-differential measurement error^3^) | Low (use FIQT) | Unclear (unclear in at least one domain) |
| Paternal smoking on maternal outcomes | Unclear (conditional F stats are small. Although ‘robust’ estimators are consistent, their SEs are very wide) | Low (No evidence of association) | Low (All GWASs adjust for PCs or use BOLT-LMM or GC) | Low (adjust for mum smoking) | Low | Low (Cochrane Q p = 0.222 | Low (data harmonised and palindromes removed when direction cannot be inferred from MAF) | Low (ALSPAC and UKB sampled form homogeneous populations. Outcome includes UKB) | Low (r^2^ of 0.001 and KB of 10,000) | Low (non-differential measurement error^3^) | Low (use FIQT) | Unclear (unclear in at least one domain) |

**Supplementary Table S3e: Risk of bias for 4 MR estimation approaches when hypertension is the outcome.**

| **MR estimation approach** | **IV1 – relevance** | **IV2 – independence** | | | | **IV3 – exclusion restriction** | **Harmonisation of SNP effects** | **Exposure-outcome GWAS from the same population^1^** | **Clumping of genetic instruments** | **Other risk of bias^2^** | **Winner’s curse** | **Overall risk** |
| --- | --- | --- | --- | --- | --- | --- | --- | --- | --- | --- | --- | --- |
|  |  | **Hair colour negative control** | **GWAS covariates or LSDC intercept.** | **Unadjusted pathways in DAG.** | **Judgment** |  |  |  |  |  |  |  |
| Maternal smoking on index individual’s outcome | Unclear (conditional F stats are small. Although ‘robust’ estimators are consistent, their SEs are very wide) | Low (No evidence of association) | Low (All GWASs adjust for PCs or use BOLT-LMM or GC) | Low (adjust for index smoking) | Low | Low (Cochrane Q p-value < 0.001, but ‘robust’ estimators provide consistent estimates) | Low (data harmonised and palindromes removed when direction cannot be inferred from MAF) | Low (exposure and outcome GWASs both include UKB) | Low (r^2^ of 0.001 and KB of 10,000) | Low (non-differential measurement error^3^) | Low (use FIQT) | Unclear (unclear in at least one domain) |
| Paternal smoking on index individual’s outcome | Unclear (conditional F stats are small. Although ‘robust’ estimators are consistent, their SEs are very wide) | Low (No evidence of association) | Low (All GWASs adjust for PCs or use BOLT-LMM or GC) | Low (adjust for index smoking) | Low | Low (Cochrane Q p-value < 0.001, but ‘robust’ estimators provide consistent estimates) | Low (data harmonised and palindromes removed when direction cannot be inferred from MAF) | Low (ALSPAC and UKB sampled form homogeneous populations. Outcome includes UKB) | Low (r^2^ of 0.001 and KB of 10,000) | Low (none) | Low (use FIQT) | Unclear (unclear in at least one domain) |
| Maternal smoking on paternal outcomes | Unclear (conditional F stats are small. Although ‘robust’ estimators are consistent, their SEs are very wide) | Low (No evidence of association) | Low (All GWASs adjust for PCs or use BOLT-LMM or GC) | Low (adjust for dad smoking) | Low | Low (Cochrane Q p = 0.723) | Low (data harmonised and palindromes removed when direction cannot be inferred from MAF) | Low (ALSPAC and UKB sampled form homogeneous populations. Outcome includes UKB) | Low (r^2^ of 0.001 and KB of 10,000) | Low (non-differential measurement error^3^) | Low (use FIQT) | Unclear (unclear in at least one domain) |
| Paternal smoking on maternal outcomes | Unclear (conditional F stats are small. Although ‘robust’ estimators are consistent, their SEs are very wide) | Low (No evidence of association) | Low (All GWASs adjust for PCs or use BOLT-LMM or GC) | Low (adjust for mum smoking) | Low | Low (Cochrane Q p-value = 0.009, but ‘robust’ estimators provide consistent estimates) | Low (data harmonised and palindromes removed when direction cannot be inferred from MAF) | Low (ALSPAC and UKB sampled form homogeneous populations. Outcome includes UKB) | Low (r^2^ of 0.001 and KB of 10,000) | Low (non-differential measurement error^3^) | Low (use FIQT) | Unclear (unclear in at least one domain) |

**Supplementary Table S3f: Risk of bias for 4 MR estimation approaches when depression is the outcome.**

| **MR estimation approach** | **IV1 – relevance** | **IV2 – independence** | | | | **IV3 – exclusion restriction** | **Harmonisation of SNP effects** | **Exposure-outcome GWAS from the same population^1^** | **Clumping of genetic instruments** | **Other risk of bias^2^** | **Winner’s Curse** | **Overall risk** |
| --- | --- | --- | --- | --- | --- | --- | --- | --- | --- | --- | --- | --- |
|  |  | **Hair colour negative control** | **GWAS covariates or LSDC intercept** | **Unadjusted pathways in DAG** | **Judgment** |  |  |  |  |  |  |  |
| Maternal smoking on index individual’s outcome | Unclear (conditional F stats are small. Although ‘robust’ estimators are consistent, their SEs are very wide) | Low (No evidence of association) | Low (All GWASs adjust for PCs or use BOLT-LMM or GC) | Low (adjust for index smoking) | Low | Low (Cochrane Q p-value < 0.001, but ‘robust’ estimators provide consistent estimates) | Low (data harmonised and palindromes removed when direction cannot be inferred from MAF) | Low (exposure and outcome GWASs both include UKB) | Low (r^2^ of 0.001 and KB of 10,000) | Low (non-differential measurement error^3^) | Low (use FIQT) | Unclear (unclear in at least one domain) |
| Paternal smoking on index individual’s outcome | Unclear (conditional F stats are small. Although ‘robust’ estimators are consistent, their SEs are very wide) | Low (No evidence of association) | Low (All GWASs adjust for PCs or use BOLT-LMM or GC) | Low (adjust for index smoking) | Low | Low (Cochrane Q p-value < 0.001, but ‘robust’ estimators provide consistent estimates) | Low (data harmonised and palindromes removed when direction cannot be inferred from MAF) | Low (ALSPAC and UKB sampled form homogeneous populations. Outcome includes UKB) | Low (r^2^ of 0.001 and KB of 10,000) | Low (none) | Low (use FIQT) | Unclear (unclear in at least one domain) |
| Maternal smoking on paternal outcomes | Unclear (conditional F stats are small. Although ‘robust’ estimators are consistent, their SEs are very wide) | Low (No evidence of association) | Low (All GWASs adjust for PCs or use BOLT-LMM or GC) | Low (adjust for dad smoking) | Low | Low (Cochrane Q p = 0.149) | Low (data harmonised and palindromes removed when direction cannot be inferred from MAF) | Low (ALSPAC and UKB sampled form homogeneous populations. Outcome includes UKB) | Low (r^2^ of 0.001 and KB of 10,000) | Low (non-differential measurement error^3^) | Low (use FIQT) | Unclear (unclear in at least one domain) |
| Paternal smoking on maternal outcomes | Unclear (conditional F stats are small. Although ‘robust’ estimators are consistent, their SEs are very wide) | Low (No evidence of association) | Low (All GWASs adjust for PCs or use BOLT-LMM or GC) | Low (adjust for mum smoking) | Low | Low (Cochrane Q p = 0.089) | Low (data harmonised and palindromes removed when direction cannot be inferred from MAF) | Low (ALSPAC and UKB sampled form homogeneous populations. Outcome includes UKB) | Low (r^2^ of 0.001 and KB of 10,000) | Low (non-differential measurement error^3^) | Low (use FIQT) | Unclear (unclear in at least one domain) |

^1^ All samples were drawn from demographically similar GWASs. Specifically, all the exposure GWAS scans were derived from either the UKB or ASLAPC. The fixation index for ASLAPC and UKB is less than 0.001 implying that they are from a homogeneous population. All outcome GWASs included data from the UKB and demographically similar (based on age, sex, and ethnicity) and can therefore be regarded as being sampled from similar populations.

^2^ Including measurement error, selection bias, failure of the positive control for the outcome (i.e. that an individual’s smoking causes the outcome in the individuals), or evidence of bias due to genotyping chip (for which there was none found – see Supplementary Tables S4).

^3^ Proxying maternal smoking using maternal smoking at birth may introduce non-differential measurement error because many mothers stop smoking during pregnancy, despite otherwise smoking. This is unlikely to produce a false positive because a) MR is robust to non-differential measurement error (1); b) outcome related differential measurement error in the exposures biases point estimates only under the alternative hypothesis (2), however point estimation is not the primary aim of our study.

**References**

1. Pierce BL, VanderWeele TJ. The effect of non-differential measurement error on bias, precision and power in Mendelian randomization studies. International Journal of Epidemiology. 2012 Oct 1;41(5):1383–93.

2. Woolf B, Karhunen V, Yarmolinsky J, Tilling K, Gill D. Re-evaluating the robustness of Mendelian randomisation to measurement error [Internet]. medRxiv; 2022 [cited 2022 Oct 5]. p. 2022.10.02.22280617. Available from: https://www.medrxiv.org/content/10.1101/2022.10.02.22280617v1
