# Supplementary material for: Assessing the causal effects of environmental tobacco smoke exposure: a meta-analytic Mendelian randomization study
Source: Nicotine Tob Res. 2026 Feb 25;28(8):1293–303. doi: 10.1093/ntr/ntag047 (PMC13389530; doi:10.1093/ntr/ntag047)
Supplement: Supplementary_Material_ntag047 [file supplementary_material_ntag047.zip › PS_Supplementary_Table_S4_ntag047.docx]

Supplementary Table S4: Difference in chip adjusted and non-chip adjusted (IVW) MR estimates

| **Description** | **Outcome** | **Chip beta** | **SE of chip beta** | **No chip beta** | **SE no chip beta** | **Difference** | **SE of difference** | **p-value of difference** |
| --- | --- | --- | --- | --- | --- | --- | --- | --- |
| dad smoking -> index | lung cancer | 0.154 | 0.241 | 0.024 | 0.077 | 0.130 | 0.253 | 0.608 |
| dad smoking -> mum | lung cancer | 1.104 | 0.673 | 0.077 | 0.142 | 1.027 | 0.688 | 0.135 |
| mum smoking -> dad | lung cancer | 2.299 | 0.548 | 1.324 | 0.382 | 0.975 | 0.668 | 0.145 |
| mum smoking -> index | lung cancer | 0.626 | 1.110 | 0.410 | 1.144 | 0.216 | 1.594 | 0.892 |
| dad smoking -> index | hypertension | -0.117 | 0.078 | -0.030 | 0.028 | -0.087 | 0.083 | 0.294 |
| dad smoking -> mum | hypertension | -0.103 | 0.253 | -0.047 | 0.070 | -0.056 | 0.263 | 0.833 |
| mum smoking -> dad | hypertension | -0.137 | 0.216 | -0.147 | 0.213 | 0.010 | 0.303 | 0.973 |
| mum smoking -> index | hypertension | -0.231 | 0.433 | -0.296 | 0.452 | 0.065 | 0.626 | 0.917 |
| dad smoking -> index | COPD | 0.125 | 0.137 | 0.032 | 0.045 | 0.093 | 0.144 | 0.519 |
| dad smoking -> mum | COPD | 0.637 | 0.349 | 0.262 | 0.190 | 0.375 | 0.397 | 0.345 |
| mum smoking -> dad | COPD | 1.722 | 0.369 | 1.171 | 0.317 | 0.550 | 0.487 | 0.258 |
| mum smoking -> index | COPD | 0.479 | 0.725 | 0.290 | 0.728 | 0.190 | 1.027 | 0.853 |
| dad smoking -> index | depression | -0.039 | 0.075 | -0.017 | 0.027 | -0.023 | 0.080 | 0.778 |
| dad smoking -> mum | depression | 0.678 | 0.429 | 0.187 | 0.120 | 0.491 | 0.445 | 0.270 |
| mum smoking -> dad | depression | 0.220 | 0.418 | 0.215 | 0.414 | 0.005 | 0.588 | 0.994 |
| mum smoking -> index | depression | -0.017 | 0.415 | 0.069 | 0.437 | -0.086 | 0.603 | 0.887 |
| dad smoking -> index | stroke | 0.140 | 0.067 | 0.053 | 0.025 | 0.087 | 0.072 | 0.226 |
| dad smoking -> mum | stroke | -0.276 | 0.255 | -0.082 | 0.071 | -0.194 | 0.265 | 0.463 |
| mum smoking -> dad | stroke | -0.078 | 0.231 | -0.087 | 0.228 | 0.010 | 0.324 | 0.975 |
| mum smoking -> index | stroke | -0.194 | 0.381 | -0.246 | 0.396 | 0.052 | 0.550 | 0.925 |
| dad smoking -> index | CHD | 0.092 | 0.072 | 0.028 | 0.026 | 0.063 | 0.076 | 0.407 |
| dad smoking -> mum | CHD | -0.295 | 0.253 | -0.096 | 0.070 | -0.199 | 0.262 | 0.448 |
| mum smoking -> dad | CHD | -0.047 | 0.182 | -0.046 | 0.180 | -0.001 | 0.256 | 0.996 |
| mum smoking -> index | CHD | 0.174 | 0.397 | 0.152 | 0.414 | 0.022 | 0.573 | 0.969 |

COPD = chronic obstructive pulmonary disease. CHD = coronary heart disease.
