# Supplementary material for: Assessing the causal effects of environmental tobacco smoke exposure: a meta-analytic Mendelian randomization study
Source: Nicotine Tob Res. 2026 Feb 25;28(8):1293–303. doi: 10.1093/ntr/ntag047 (PMC13389530; doi:10.1093/ntr/ntag047)
Supplement: Supplementary_Material_ntag047 [file supplementary_material_ntag047.zip › PS_Supplementary_Table_S5_MM_bw_ntag047.docx]

**Supplementary Table S5: Results of hair colour negative control outcome analysis.**

| **Exposure** | **Outcome** | **Number of SNPS** | **IVW estimate** | **Standard error** | | **p-value** |
| --- | --- | --- | --- | --- | --- | --- |
| index smoking | Blond | 120 | 0.005 | | 0.014 | 0.734 |
| index smoking | Red | 120 | -0.034 | | 0.044 | 0.432 |
| index smoking | Light brown | 120 | 0.016 | | 0.010 | 0.114 |
| index smoking | Dark brown | 120 | 0.004 | | 0.045 | 0.932 |
| index smoking | Black | 120 | 0.006 | | 0.009 | 0.522 |
| index smoking | Other | 120 | 0.002 | | 0.003 | 0.477 |
| Paternal smoking | Blond | 4 | 0.012 | | 0.013 | 0.360 |
| Paternal smoking | Red | 4 | 0.006 | | 0.011 | 0.568 |
| Paternal smoking | Light brown | 4 | -0.040 | | 0.020 | 0.051 |
| Paternal smoking | Dark brown | 4 | 0.002 | | 0.020 | 0.931 |
| Paternal smoking | Black | 4 | 0.014 | | 0.008 | 0.093 |
| Paternal smoking | Other | 4 | 0.006 | | 0.007 | 0.392 |
| Maternal smoking | Blond | 16 | -0.005 | | 0.029 | 0.858 |
| Maternal smoking | Red | 16 | -0.007 | | 0.019 | 0.714 |
| Maternal smoking | Light brown | 16 | 0.015 | | 0.062 | 0.815 |
| Maternal smoking | Dark brown | 16 | 0.001 | | 0.064 | 0.990 |
| Maternal smoking | Black | 16 | -0.009 | | 0.018 | 0.629 |
| Maternal smoking | Other | 16 | 0.006 | | 0.010 | 0.583 |

The only MR estimator used here was the inverse variance waited estimator on the grounds that a ‘pleiotropic’ association of the genetic instruments with hair colour could still indicate residual population structure.
