# Supplementary material for: Assessing the causal effects of environmental tobacco smoke exposure: a meta-analytic Mendelian randomization study
Source: Nicotine Tob Res. 2026 Feb 25;28(8):1293–303. doi: 10.1093/ntr/ntag047 (PMC13389530; doi:10.1093/ntr/ntag047)
Supplement: Supplementary_Material_ntag047 [file supplementary_material_ntag047.zip › PS_Supplementary_Table_S6_ntag047.docx]

| **Putative Explanation** | **Description** | **Can it account for specificity of extreme effects with respect to the maternal smoking on maternal outcome design (i.e. why the paternal effects are not extreme)** | **Can it account for specificity of extreme effects with respect to outcomes (i.e. why only respiratory outcomes have such large effects)** |
| --- | --- | --- | --- |
| Recall bias in maternal smoking (outcome related differential measurement error) | Individuals whose parents had lung cancer or COPD might more accurately remember if their mother smoked when they were children | Yes: Paternal and index individual smoking use prospective and/or objectively measured which should protect against recall bias. | Yes: parental lung cancer and COPD have much better known connections to parental smoking than the other outcomes, protecting the other outcomes from recall bias. Offspring outcomes were also measured objectively using medical record codes. |
| Frist-hand smoking and ETS estimates are not comparable. | a measured SD exposure to ETS represents less variability in smoke inhalation than a SD of first-hand smoke. For example, how someone smokes (such as how deeply someone breaths in first-hand smoke) will create variability in first-hand smoking which is not present for ETS which to a greater either is or is not present. | No. This explanation implies that similar observations should be seen for all outcomes in all maternal smoking analyses. | |
| There is plausible age and duration of exposure effect modification | Smoking effects on COPD and lung cancer are known to follow a dose response effect. Parents can be exposed to each others smoking for longer than UKB participants have been alive which might explain the larger effect. | Yes: The generation of parents of UKB participants should have larger effects because the parents will be older and exposed to more of each other’s smoke than their offspring. | No: other outcomes that also have dose-response relationships with smoking are not inflated. |
| An assessable violations of the core IV assumptions | A violation of the core IV assumptions could produce biased estimates | No: The sensitivity analyses (see Supplementary Table S2 to 5) present no evidence that these analyses are at specific risk of bias due to a violation of the IV assumptions, and therefore cannot explain why the extreme results are specific these answers. | |
| An unknown design flaw (e.g. an unmeasured or residual source of bias) | Observing larger effects than for first hand smoking might represent a general failure of the design to account for offspring mediated effects or some other systematic source or bias. | No: While this hypothesis cannot be disproved, it provides no reason why the effects would be specific to these analyses. For example, unmeasured pleiotropy or residual effects due to index/paternal smoking should be similar for both maternal smoking designs and all outcomes. | |

Supplementary Table S6: discussion of alternative explanations for the extreme effects observed for maternal smoking and paternal respiratory outcomes
